# Transparency, accountability, and scholarly integrity: A systematic narrative review of artificial intelligence governance frameworks in healthcare publication

**DOI:** 10.1371/journal.pdig.0001783

**Published:** 2026-10-20

**Authors:** Amina Silva, Bruna Canever, Monica Lino, Vanessa Silva e Silva

**Affiliations:** 1 School of Nursing, Brock University, St Catharines, Canada; 2 Nursing Department, Federal University of Santa Catarina, Florianopolis, Brazil; 3 School of Nursing, University of Central Florida, Orlando, Florida, United States of America; Tehran University of Medical Sciences, IRAN, ISLAMIC REPUBLIC OF

## Abstract

The integration of artificial intelligence (AI) in healthcare research and scholarly publication has produced a rapidly expanding but fragmented ecosystem of reporting guidelines, governance frameworks, editorial policies, and regulatory instruments, imposing significant burdens on researchers, reviewers, and editors. This systematic narrative review identified, stratified, and synthesized governance frameworks and reporting standards addressing transparent and ethical AI use in healthcare publications, and characterized the governance trajectory from predictive model standards to generative AI oversight. Fourteen Boolean search strings were executed in PubMed (MEDLINE) covering 1 January 2018–19 May 2026, supplemented by manual searching of the EQUATOR Network guideline library. Records were deduplicated and prioritized using a relevance-scoring algorithm. Of 30,276 deduplicated records, 888 progressed to human screening; 100 publications were included, comprising 69 governance instruments and policies classified across four evidence tiers and 31 contextual evidence sources. Included sources spanned six thematic groups: predictive and diagnostic AI frameworks (G1); sociotechnical and lifecycle governance (G2); generative AI and LLM-specific standards (G3); specialty-specific extensions (G4); surgical reporting updates (G5); and editorial and publication policy (G6). Analysis identified four consecutive phases of governance development from 2018 to 2026, characterized by a shift from model performance transparency toward comprehensive sociotechnical accountability. Three governance tensions emerged: the trade-off between framework specificity and universal applicability; the gap between disclosure-focused standards and lifecycle governance; and the risk of epistemic conflation, whereby heterogeneous instruments are perceived as carrying equivalent authority when treated as interchangeable. AI governance in healthcare publications remains fragmented and epistemically uneven. Advancing the field requires harmonization across evidence tiers, adaptive updating mechanisms, and lifecycle-oriented governance frameworks.

## Author summary

Artificial intelligence is increasingly used in healthcare research, from developing clinical tools to helping prepare scientific papers. At the same time, many guidelines and policies have been created to tell researchers, journals, and reviewers how this work should be reported and managed. We reviewed this rapidly growing landscape to understand what guidance exists, how it has changed over time, and where important gaps remain. We identified 100 relevant publications, including 69 guidelines, frameworks, and institutional or editorial policies. We found that guidance has evolved from a strong focus on how artificial intelligence models perform toward broader concerns such as fairness, transparency, human oversight, and the use of generative artificial intelligence in research and publishing. However, the current landscape remains fragmented. Different documents vary greatly in how they were developed and how much authority they carry, while many focus mainly on disclosure when a paper is published rather than responsibilities throughout the life of an artificial intelligence system. Our findings suggest that the field now needs better coordination of existing guidance, clearer direction on which standards should be used in different situations, and stronger attention to responsibilities beyond publication.

## 1. Introduction

The use of artificial intelligence (AI) in healthcare research and scholarly publication has expanded substantially, spanning manuscript drafting, literature synthesis, data analysis, peer review, and clinical prediction modelling [1,2]. This expansion has prompted the development of a large and heterogeneous body of governance instruments, including reporting guidelines, editorial policies, appraisal tools, governance frameworks, and regulatory standards. The result is an ecosystem that is simultaneously expansive and fragmented: researchers must navigate dozens of overlapping instruments across study designs, specialties, and publication contexts, without a clear hierarchy of evidence or applicability [3,4].

Existing reviews of AI reporting guidelines have been limited in scope, typically focusing on narrow methodological families, such as predictive modelling [5,6], or on single domains, such as radiology [7]. None has systematically stratified the governance ecosystem by evidence tier, nor has any captured the rapid 2025–2026 expansion of LLM-specific frameworks. Furthermore, existing reviews seldom engage with the conceptual tensions underlying governance fragmentation, particularly the trade-off between framework specificity and universal applicability and the gap between disclosure-oriented standards and lifecycle accountability.

This review addresses three specific gaps. First, it systematically identifies governance frameworks and reporting standards across study designs, specialties, and publication contexts captured through the predefined search strategy. Second, it stratifies included instruments by evidence tier to avoid treating editorial policies and EQUATOR-listed guidelines as epistemically equivalent. Third, it identifies the governance tensions that currently constrain harmonization. The objective was to identify, stratify, and synthesize governance frameworks and reporting standards addressing transparent and ethical AI use in healthcare publications, and to characterize the governance trajectory from predictive model standards to generative AI oversight.

### 1.1 Research question

What governance frameworks and reporting standards currently address transparent and ethical AI use in healthcare publications?How do they stratify by evidence tier and thematic group, and what governance tensions emerge from their synthesis?

## 2. Methods

### 2.1 Review design

A systematic narrative review [8] was conducted and reported in accordance with the PRISMA 2020 statement [9]. This design was selected because the objective required comprehensive mapping and thematic synthesis across a heterogeneous body of governance instruments, including non-empirical policy documents, editorial statements, and proposed frameworks, which do not lend themselves to meta-analysis or formal quality appraisal. All screening, eligibility assessment, and interpretive synthesis were performed by the research team.

### 2.2 Search strategy and screening

Fourteen pre-specified Boolean search strings were executed in PubMed (MEDLINE) with final search in May 2026, covering January 2018 to May 2026. Search blocks addressed: (1) core AI reporting; (2) publication ethics and AI; (3) named EQUATOR-registered guidelines; (4) systematic reviews of AI guidelines; (5) predictive model reporting; (6) LLM and chatbot reporting; (7) radiology and imaging AI; (8) specialty-specific frameworks; (9) fairness and bias reporting; (10) health economics; (11) editorial and publisher policies; (12) real-world evidence; (13) clustered prediction models; and (14) AI governance and regulation. The EQUATOR Network guideline library (equator-network.org) was also manually searched. A full list of Boolean strings is provided in S1 Table.

Following the search and removal of duplicates, a relevance-scoring algorithm that assigned scores based on the presence of pre-specified domain terms in the title and abstract prioritized 888 records for human screening; the remaining 29,388 records scored below the threshold and were not individually assessed. Records identified through the EQUATOR manual search were entered into the same deduplication and relevance-scoring workflow as the PubMed records and were subject to the same threshold, with no exemption on the basis of source. The algorithm and scoring variables are described in the S2 Table. We acknowledge that records scoring below the threshold may include relevant instruments; this represents an inherent limitation of algorithmic pre-screening. The search was limited to PubMed and EQUATOR; Scopus, Web of Science, and grey literature beyond EQUATOR were not searched, which may have resulted in under-representation of policy documents and non-English sources. Full PRISMA record counts are reported in S3 Table.

The relevance-scoring algorithm assigned scores using 16 pre-specified inclusion signal terms (each contributing +1 point), 15 exclusion signal terms (each contributing −2 points), and a named-acronym bonus (+2 points if any of 19 named guideline acronyms appeared in the record title). Scores were floored at zero; no maximum cap was applied. Records scoring ≥3 were designated high-priority and proceeded to human title and abstract screening; records scoring <3 were not advanced and were not individually assessed. The threshold of ≥3 was assessed during pilot testing against a set of previously identified relevant records. A formal validation of the algorithm against an independent reference set of relevant records and a representative sample of below-threshold records was not undertaken for this revision; consequently, the algorithm’s sensitivity and its false-negative rate below the threshold have not been empirically quantified. This is acknowledged as a methodological limitation, and the potential for relevant records scoring below the threshold to have been missed is addressed explicitly in the Limitations. Records identified through the EQUATOR Network manual search (n = 22) were combined with PubMed records, deduplicated, and scored using the same relevance-scoring function as all other records. Full documentation of the algorithm is provided in S2 Table; the implementing Python script is in S5 Table.

### 2.3 Eligibility criteria

Documents were included if they: (1) presented guidance, recommendations, reporting standards, editorial policies, or governance considerations specifically addressing AI or machine learning use in healthcare research or scientific dissemination; (2) addressed healthcare, medicine, nursing, public health, or biomedical science; and (3) provided actionable reporting items, structured recommendations, or defined governance principles. Documents were excluded if they: (1) addressed technical AI development without implications for research reporting, publication, dissemination, or governance; (2) discussed AI ethics or policy without actionable reporting or governance guidance; (3) did not address healthcare, medicine, nursing, public health, or biomedical science; or (4) represented duplicates, superseded versions, or non-distinct versions of instruments already included. Eligibility decisions were made by reviewers (A.S.) for all 888 records advancing to this stage, and conflicts were solved with another reviewer (V.S.S.). Exclusion reasons during human screening were recorded at the category level rather than at the individual publication level.

### 2.4 Data synthesis

Descriptive synthesis characterized included sources by publication year, type, and geographic origin. Narrative synthesis applied content analysis to identify thematic groupings, governance phases, and cross-cutting tensions. Consistent with the design of a systematic narrative review, formal quality appraisal of individual sources was not performed; instead, evidence tier assignment describes developmental maturity and governance authority rather than serving as a quality signal, and is not a substitute for formal quality appraisal. Where a predefined extraction field was not reported in a source, it was coded as “not reported.” Where a field did not apply to the document type, it was coded as “not applicable.” No values were imputed and no unreported information was inferred.

The six thematic groups and four governance phases were derived inductively within this review: as each of the 100 included publications was extracted and characterized, it was mapped to an emergent framework category based on its primary governance function, target study design, and institutional origin. Initial groupings were proposed by the lead author, reviewed iteratively by co-authors during the synthesis phase, and finalized by team consensus. Where a source addressed frameworks spanning multiple groups, it was assigned to the group corresponding to its primary governance focus. The four governance phases were identified through chronological content analysis: all 100 sources were ordered by publication year and reviewed for shifts in governance priorities, instrument type, and institutional origin. Phase boundaries were identified by the lead author and finalized by co-author consensus.

Tier assignment process: Tier criteria were pre-specified before data extraction. Assignment was performed by the lead author (A.S.) and verified by a second author (V.S.S.) for all 100 sources. Disagreements were resolved through consensus discussion among the full author team. Formal inter-rater reliability statistics were not calculated, which we acknowledge as a limitation; the pre-specified, criterion-referenced nature of the tier definitions, particularly the objective Tier 1 criterion of EQUATOR registration, substantially reduced the degree of interpretive judgement required for the majority of assignments. Borderline cases were resolved through team consensus and are noted in S4 Table.

### 2.5 Evidence stratification

A key methodological contribution of this review is explicit classification of included instruments by evidence tier. The tiers describe developmental maturity and governance authority, derived from development pathway, publication status, and institutional provenance, and do not measure methodological quality, validity, representativeness, or implementability; they are not a substitute for formal quality appraisal. We applied the following hierarchy: Tier 1, Formal EQUATOR-listed reporting guidelines: developed through defined expert consensus (typically Delphi), registered on the EQUATOR Network, and published in peer-reviewed journals. These carry the strongest governance authority within the reporting-guideline ecosystem. Tier 2, Peer-reviewed consensus frameworks: published in indexed journals using systematic consensus methods, but not formally EQUATOR-registered or developed under EQUATOR governance. Tier 3, Emerging, specialty, or proposed instruments: published but preliminary (described as ‘pilot’ or ‘proposed’), or developed by individual journal editorial teams without multi-stakeholder consensus. These warrant cautious interpretation pending further validation. Tier 4, Editorial, institutional, and regulatory policies: statements from professional organizations, publishers, funding bodies, or regulatory authorities. Authoritative within their jurisdictions but not designed as methodological appraisal instruments. This stratification is carried through Table 1 and the narrative synthesis. When citing specific frameworks, we note their tier explicitly to avoid epistemic conflation.

**Table 1:** Abbreviated overview of named AI reporting and governance frameworks by thematic group and evidence tier (n = 42; full characteristics in S4 Table).

Group	Framework	Scope	Year	Key reference	EQUATOR	Tier
**G1 — Predictive and Diagnostic AI Frameworks (n = 9 named frameworks)**						
G1	CONSORT-AI	RCT reporting for AI interventions	2020	Liu et al. (2020). BMJ, 370, m3164.	Yes	Tier 1
G1	SPIRIT-AI	AI clinical trial protocols	2020	Cruz Rivera et al. (2020). Lancet Digit Health.	Yes	Tier 1
G1	TRIPOD+AI	ML prediction model reporting	2024	Collins et al. (2024). BMJ, 385, e078378.	Yes	Tier 1
G1	PROBAST+AI	Risk of bias in AI prediction models	2025	Moons et al. (2025). BMJ, 388, e082505.	Yes	Tier 1
G1	STARD-AI	AI diagnostic accuracy reporting	2021/2025	Sounderajah et al. (2025). Nat Med, 31(10), 3283–3289.	Yes	Tier 1
G1	CLAIM/ 2024 update	Medical imaging AI research	2020/2024	Mongan et al. (2020); Tejani et al. (2024).	Yes	Tier 1
G1	QUADAS-AI	Risk of bias in AI diagnostic accuracy reviews	2024	Guni et al. (2024). JMIR Res Protoc.	No	Tier 3
G1	CHEERS-AI	Health economic evaluation of AI interventions	2024	Elvidge et al. (2024). Value in Health.	Yes	Tier 1
G1	MI-CLAIM	Minimum information for clinical AI modelling	2020	Norgeot et al. (2020). Nat Med.	Yes	Tier 1
**G2 — Sociotechnical and Lifecycle Governance (n = 5 named frameworks)**						
G2	DECIDE-AI	Early-stage clinical AI evaluation	2022	Vasey et al. (2022). Nat Med, 28, 924.	Yes	Tier 1
G2	FUTURE-AI	Trustworthy AI across the full lifecycle	2023/2025	Lekadir et al. (2025). BMJ, 388, e081554.	No	Tier 2
G2	STANDING Together (2023/2025)	Algorithmic bias and health dataset diversity	2023/2025	Alderman et al. (2023). Lancet Digit Health.	No	Tier 2
G2	AIHGIe 2.0	Transparent AI development and reporting governance	2026	Ministry of Health & Health Sciences Authority, Singapore (2026).	No	Tier 4
G2	TREG-AI	Ethical reporting of generative AI research	2023	Liu et al. (2023). eClinicalMedicine.	No	Tier 3
**G3 — Generative AI and LLM-Specific Standards (n = 9 named frameworks)**						
G3	GAMER	Generative AI use in medical research	2025	Luo et al. (2025). BMJ Evid Based Med.	Yes	Tier 1
G3	CHART	Chatbot health advice evaluation	2025	The CHART Collaborative (2025). JAMA Netw Open et al.	Yes	Tier 1
G3	TRIPOD-LLM	LLM development and evaluation studies	2025	Gallifant et al. (2025). Nat Med, 31, 60.	Yes	Tier 1
G3	CANGARU	GenAI use in academic research and publication	2023	Cacciamani et al. (2023). arXiv:2307.08974.	Under dev.	Tier 3
G3	REFINE	Foundation models and LLMs in medical research	2026	Mese et al. (2026). Diagn Interv Radiol.	No	Tier 2
G3	MEDAI-LLM-SUMM	LLM medical text summarisation	2026	Khoruzhaya et al. (2026). Front Digit Health.	No	Tier 2
G3	MI-CLEAR-LLM (2025 update)	LLM accuracy reporting in healthcare	2025	Park et al. (2025). Korean J Radiol.	No	Tier 3
G3	PRISMA-trAIce	AI use in systematic literature reviews	2025	Gottlieb et al. (2025). JMIR AI.	No	Tier 3
G3	COREQ+LLM	LLM-mediated qualitative research	2025	Fehring et al. (2025). JMIR Res Protoc.	No	Tier 3
**G4 — Specialty-Specific AI Frameworks (n = 9 named frameworks)**						
G4	PRIME/ PRIME 2.0	Cardiovascular imaging ML/multimodal AI	2020/2026	Sengupta et al. (2020); Kagiyama et al. (2026). JACC Cardiovasc Imaging.	Yes	Tier 2
G4	STREAM-URO	ML reporting in urology	2021	Kwong et al. (2021). Eur Urol Focus.	Yes	Tier 2
G4	R-AI-DIOLOGY	AI appraisal in clinical neuroradiology	2022	Haller et al. (2022). Neuroradiology.	No	Tier 2
G4	CLEAR Derm †	Image-based AI in dermatology	2022	Daneshjou et al. (2022). JAMA Dermatol.	No	Tier 2
G4	CLEAR Radiomics †	Radiomics research evaluation (ESR/EuSoMII)	2023	Kocak et al. (2023). Insights Imaging.	Yes	Tier 1
G4	METRICS Radiomics ‡	Radiomics quality scoring (EuSoMII)	2024	Kocak et al. (2024). Insights Imaging.	Yes	Tier 1
G4	CAIR	General clinical AI research appraisal	2021	Olczak et al. (2021). Acta Orthop.	Yes	Tier 3
G4	FLAIR	Framework for LLM Assessment in Radiology (international Delphi)	2026	Kottlors et al. (2026). Radiology.	No	Tier 2
G4	GRAITE-USRA	AI evaluations for ultrasound scanning in regional anaesthesia	2025	Zhang et al. (2025). Anaesthesia.	No	Tier 2
G5* — Surgical and Procedural Reporting Updates (n = 3 named frameworks)						
G5	SCARE 2025	Surgical case reports with AI update	2025	Kerwan et al. (2025). PJS.	Yes	Tier 1
G5	PROCESS 2025	Surgical case series with AI update	2025	Agha et al. (2025). PJS.	Yes	Tier 1
G5	STROCSS 2025	Surgical cohort and observational studies with AI	2025	Agha et al. (2025). PJS.	Yes	Tier 1
**G6 — Editorial and Publication Policy (n = 7 named frameworks)**						
G6	ICMJE/ WAME policies	AI authorship prohibition and disclosure standards	2023/2024	ICMJE (2024); Zielinski et al. (2023).	—	Tier 4
G6	COPE position statement	Editorial decision-making ethics for AI	2021/2023	COPE Council (2023).	—	Tier 4
G6	NIH policies (2023/2025)	AI in grant applications and peer review	2023/2025	NIH NOT-OD-23-149; NOT-OD-25-132.	—	Tier 4
G6	WHO guidance (2024)	Global ethics for large multi-modal AI models in health	2024	World Health Organization (2024).	—	Tier 4
G6	MINIMAR	Minimum information for medical AI reporting	2020	Hernandez-Boussard et al. (2020). JAMIA.	Yes	Tier 3
G6	KJA Comprehensive Checklist	Universal design-agnostic AI reporting checklist	2025	Kwak & Kim (2025). Korean J Anesthesiol.	No	Tier 3
G6	REFORMS	ML-based scientific claims (cross-disciplinary)	2024	Kapoor et al. (2024). Sci Adv.	Yes	Tier 1

† CLEAR refers to two distinct instruments: CLEAR Derm (dermatology, Tier 2) and CLEAR Radiomics (ESR/EuSoMII-endorsed, Tier 1). Manuscripts citing either must disambiguate explicitly. ‡ METRICS refers to two distinct instruments: METRICS Radiomics (quality scoring tool, Tier 1) and METRICS Generative AI checklist (JMIR, preliminary, Tier 3). Tier 1 = EQUATOR-listed guideline developed through documented multi-stakeholder consensus; Tier 2 = peer-reviewed consensus framework not meeting all Tier 1 criteria; Tier 3 = emerging or proposed instrument pending validation; Tier 4 = editorial or institutional policy. — = not applicable.

* Group 5 is separated from Group 4 because it represents updates to established non-AI surgical case reporting standards (SCARE, PROCESS, STROCSS) rather than AI-specific instruments developed for diagnostic domains; this constitutes a qualitatively different governance activity, adapting existing procedural documentation structures to incorporate AI disclosure requirements rather than creating new AI-specific frameworks.

### 2.6 Governance domain coverage mapping (Fig 2)

Fig 2 displays the coverage of eight governance domains across the six thematic framework groups. The review team predefined the eight domains before extraction, based on domains recurring in the reporting-guideline and AI governance literature, and fixed them as extraction fields in the data-extraction form. They were: (1) model performance and validation reporting; (2) data provenance and dataset documentation; (3) fairness, bias, and equity; (4) transparency and explainability; (5) authorship, disclosure, and provenance of AI-generated content; (6) clinical implementation and human factors; (7) post-deployment monitoring and lifecycle maintenance; and (8) environmental and resource sustainability. A domain was recorded as present in an instrument when the instrument contained at least one explicit reporting item, checklist element, or stated requirement addressing that domain; general aspirational language without an associated item was recorded separately as partial coverage. The unit of analysis for extraction was the individual named framework (n = 42); the unit of display in Fig 2 is the thematic group (n = 6). Domain coverage was coded for each named framework by the lead author (A.S.) and independently verified by a second author (V.S.S.), with disagreements resolved by consensus with the full author team; formal inter-rater reliability statistics were not calculated. Group-level ratings were derived from the instrument-level codes using pre-specified decision rules: primary coverage required explicit items in more than half of the named frameworks in a group, moderate coverage required explicit items in at least one but not more than half, and minor coverage indicated partial coverage only or none. Ratings were not weighted by evidence tier. The instrument-level domain codes underlying Fig 2 are provided in S4 Table so that the group-level ratings can be reproduced.

### 2.7 Use of AI tools in the conduct and preparation of this review

The relevance-scoring algorithm used for record prioritization was implemented in Python using the pre-specified scoring rules described above; the corresponding script is provided in S5 Table. Screening, eligibility assessment, evidence-tier assignment, derivation of thematic groups and governance phases, governance-domain coding, and interpretation of findings were performed by the named authors. Grammarly, an AI-assisted writing tool, was used for language editing. All AI-assisted output was reviewed and verified by the authors, who take full responsibility for the manuscript.

## 3. Findings

Following the data analysis process (Fig 1), our results were organized by the review team into four interpretive themes: (1) search results and source characteristics; (2) framework group characteristics; (3) four phases of AI governance development; and (4) three cross-cutting governance tensions. Findings are presented in a descriptive narrative manner, but all included sources, along with their characteristics, are reported in S4 Table.

**Fig 1:**
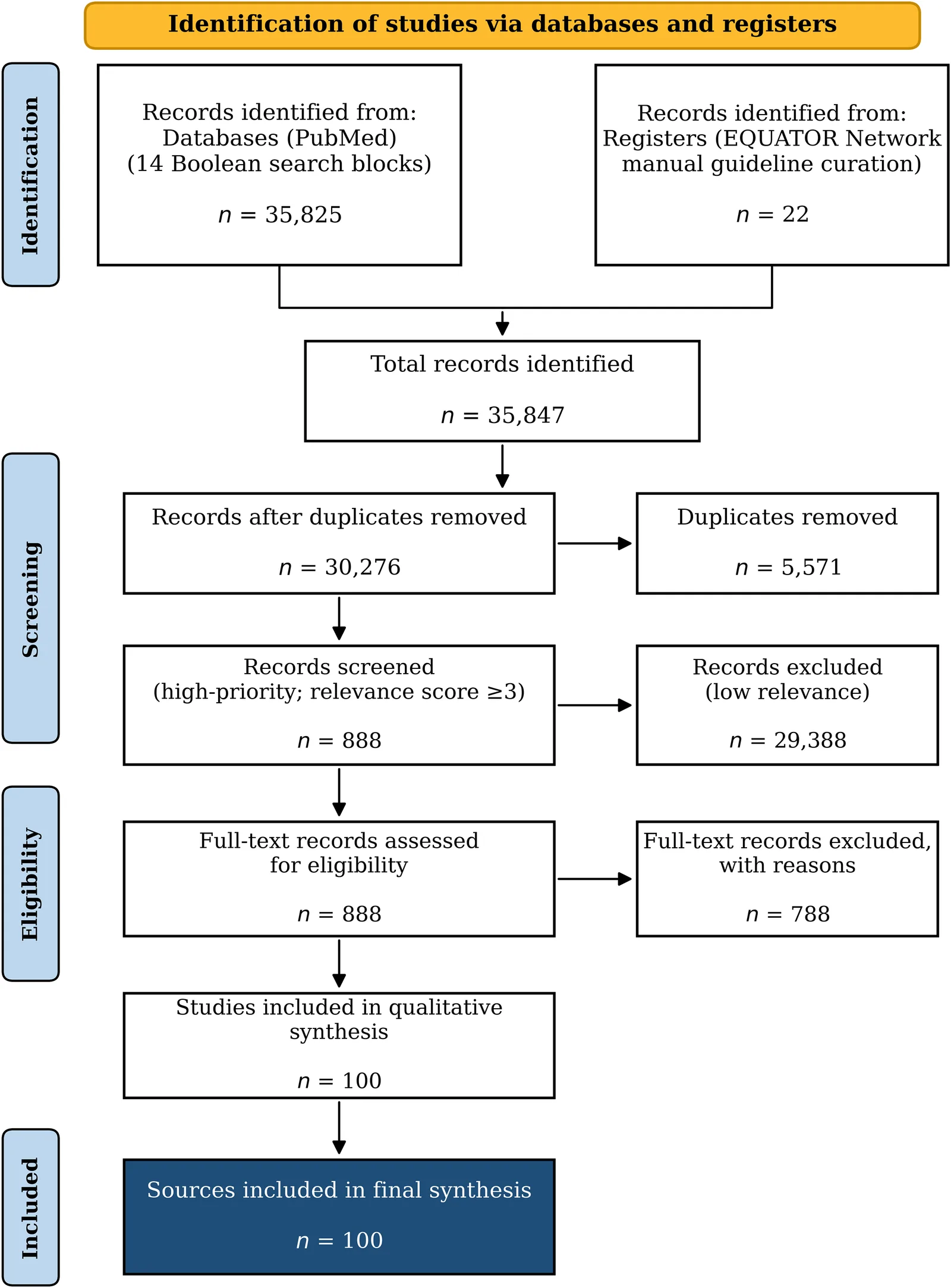
PRISMA flow diagram—Systematic search and selection process.

### 3.1 Search results and source characteristics

The 100 included publications comprised 69 governance instruments and policies and 31 contextual evidence sources used in the narrative synthesis. The 69 governance instruments and policies were stratified as follows: 20 formal EQUATOR-listed reporting guidelines (Tier 1); 12 peer-reviewed consensus frameworks developed through documented expert or Delphi processes but not EQUATOR-listed (Tier 2); 12 preliminary, proposed, or emerging instruments (Tier 3); and 25 institutional, editorial, publisher, or regulatory policies (Tier 4). The remaining 31 included publications are systematic reviews, narrative reviews, bibliometric analyses, and editorial commentaries that provided contextual evidence supporting the narrative synthesis; they are not assigned to an evidence tier. The PRISMA flow diagram of the search and selection process is shown in Fig 1. During revision, the included set was re-audited against the exclusion criteria and two records were removed as non-distinct versions of instruments already included, reducing the total from 102 in the original submission to 100; all counts, denominators, tier totals, and supporting files were updated accordingly. Three analytical denominators are used throughout and should not be conflated: the 100 included publications for all source-level statements; the 69 governance instruments and policies for all tier-stratified analyses; and the 42 named frameworks for Table 1, the thematic group counts, and Fig 2.

**Fig 2:**
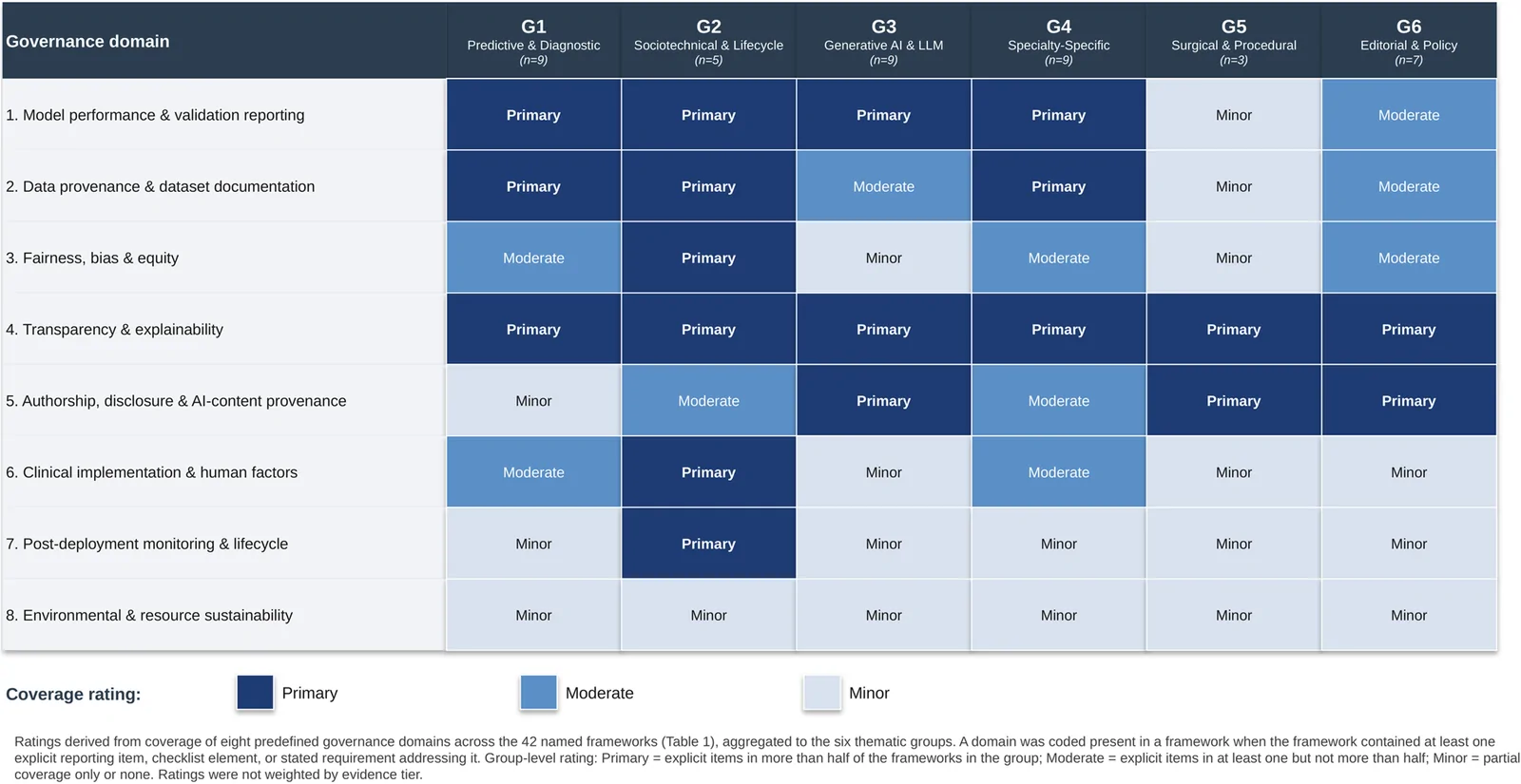
Governance domain coverage across six thematic framework groups.

Within the 69 governance instruments and policies, Tier 4 editorial and institutional policies (n = 25) constitute the largest single tier, reflecting the urgency of publisher and institutional responses to generative AI. Delphi-based guideline development (Tier 1) was predominantly led by EQUATOR-affiliated international collaborations, primarily from the United Kingdom and the United States, with growing contributions from East Asia, particularly in radiology and imaging. Nearly all included publications were in English (n = 99); one Portuguese report addressed regional policy. The paucity of non-English and non-Western sources represents a structural limitation of the current governance ecosystem, as acknowledged by the STANDING Together recommendations.

### 3.2 Framework group characteristics

Table 1 summarises the 42 named frameworks across the six thematic groups. Consistent with the governance trajectory described above, Group 1 (predictive and diagnostic, n = 9 named frameworks) contains the largest number of Tier 1 instruments. Group 3 (generative AI and LLM-specific, n = 9 frameworks) contains the highest proportion of Tier 3 instruments, reflecting the recency and preliminary status of many LLM-specific tools. Group 6 (editorial and publication policy, n = 7 named instruments) is dominated by Tier 4 statements. Group 4 (specialty-specific, n = 9 frameworks) presents notable naming conflicts, CLEAR refers to both CLEAR Derm (dermatology) and CLEAR Radiomics, and METRICS refers to both a radiomics quality score and a generative AI checklist, which require explicit disambiguation in manuscripts citing these instruments.

The six thematic groups and four governance phases reported here are an interpretive synthesis produced by the review team, not a pre-existing, externally derived, or validated taxonomy; alternative defensible groupings and boundary years are possible, and the scheme should be read as an organizing heuristic. The six groups are: (G1) Predictive and Diagnostic AI Frameworks;(G2) Sociotechnical and Lifecycle Governance; (G3) Generative AI and LLM-Specific Standards; (G4) Specialty-Specific AI Frameworks; (G5) Surgical and Procedural Reporting Updates; and (G6) Editorial and Publication Policy. Within G4, imaging-focused frameworks included PRIME and PRIME 2.0 for cardiovascular imaging, CLEAR Derm, CLEAR Radiomics, METRICS Radiomics, R-AI-DIOLOGY, and FLAIR, the radiology LLM Delphi guideline. STREAM-URO, CAIR, and GRAITE-USRA (ultrasound-guided regional anaesthesia) addressed other specialty-specific clinical contexts. These instruments are retained within one specialty-specific group because their principal distinguishing feature is adaptation to a defined clinical specialty rather than a separate governance function.

### 3.3 Four phases of AI governance development

Synthesis of the 100 included publications revealed four consecutive governance phases: (1) 2018–2022: Model-centric reporting standards; (2) 2022–2023: Sociotechnical and lifecycle governance; (3) 2023–2025: Generative AI and LLM governance; and (4) 2025–2026: Consolidation and specialty extension. These phases are not sharply bounded; frameworks in adjacent phases coexist and interact, yet they represent identifiable conceptual shifts in governance priorities.

#### 3.3.1 Phase 1 — Foundational AI reporting standards.

The first phase was dominated by formal EQUATOR-listed extensions to established reporting instruments, focused primarily on transparent reporting of predictive model performance and included publications of evidence Tiers 1–3. In the Tier 1 category CONSORT-AI and SPIRIT-AI adapted randomized trial standards to AI intervention studies; TRIPOD+AI and PROBAST+AI updated prediction model reporting and appraisal standards, respectively; and CLAIM addressed AI in medical imaging. MINIMAR (Tier 3) proposed minimum information standards. Together, these instruments established a methodological baseline for AI reporting but focused narrowly on statistical and algorithmic performance metrics, leaving sociotechnical dimensions largely unaddressed.

#### 3.3.2 Phase 2 — Sociotechnical and lifecycle governance.

A second phase broadened governance beyond manuscript reporting to encompass the full clinical AI lifecycle and also included publications of evidence Tier 1 and Tier 2. The Tier 1 DECIDE-AI introduced a reporting guideline for early-stage clinical evaluation that emphasizes human-computer interaction, workflow integration, and usability alongside performance. FUTURE-AI and STANDING Together (both Tier 2) extended governance further to address fairness, traceability, explainability, and environmental sustainability across the entire deployment lifecycle; and STANDING Together shifted attention to the upstream problem of dataset diversity and structural bias in training data. This phase marked a conceptual inflection: governance began to treat AI systems not merely as technical tools requiring performance evaluation, but as sociotechnical artifacts embedded in clinical, organizational, and ethical contexts.

#### 3.3.3 Phase 3 — Generative AI and editorial-policy expansion.

The emergence of generative AI and LLMs as mainstream research and writing tools from 2023 onward precipitated a third governance phase characterized by the rapid proliferation of editorial policies, disclosure frameworks, and LLM-specific reporting checklists. Tier 4 editorial and publication policies dominated this phase: the ICMJE, WAME, and COPE Council established the foundational prohibition against AI authorship, and major publishers, including Nature Portfolio, Elsevier, and Wiley, issued parallel Tier 4 policies aligned with these positions. Simultaneously, formal Tier 1 methodological instruments emerged to govern LLM research itself: GAMER standardized the reporting of generative AI use in medical research; CHART addressed chatbot health advice evaluation and achieved notable multi-journal simultaneous endorsement across JAMA Network Open, Artificial Intelligence in Medicine, Annals of Family Medicine, and the British Journal of Surgery; and TRIPOD-LLM extended the TRIPOD framework to LLM development and evaluation studies. The volume of Tier 4 editorial and publisher output in this phase should not be taken to imply methodological rigour; the Tier 1 instruments (GAMER, CHART, TRIPOD-LLM) are distinct from the policy statements in both their development method and their evidential authority.

#### 3.3.4 Phase 4 — Consolidation and specialty adaptation.

A fourth phase, still ongoing at the search cutoff, is characterized by two concurrent developments: the consolidation of existing frameworks into integrated tools and the proliferation of specialty-specific LLM standards, with evidence ranging from Tier 1–3 as follows. Tier 1: Surgical reporting consortia integrated AI-specific domains into established guidelines: SCARE, PROCESS, and STROCSS were each updated in 2025 to include AI configuration disclosure requirements (Tier 1). Tier 2: In radiology, FLAIR, the 2026 international Delphi-based checklist for LLM reporting (Tier 2), addressed the specific reproducibility and safety challenges of LLM deployment in imaging workflows. PRIME 2.0 (Tier 2) updated the cardiovascular imaging framework for multimodal AI. REFINE (Tier 2) and MEDAI-LLM-SUMM (Tier 2) provide targeted consensus-based checklists for foundation model reporting and medical text summarisation, respectively. Tier 3: The 2025 update of MI-CLEAR-LLM (Tier 3) provides minimum reporting items for LLM accuracy studies and should be used with the understanding that formal endorsement and validation are pending. PRISMA-trAIce (Tier 3) extended PRISMA guidance to systematic reviews using AI assistance.

### 3.4 Cross-cutting governance tensions

Synthesis across the four phases of AI governance development reveals three governance tensions that recur across framework groups and evidence tiers, and which help explain why fragmentation persists despite framework proliferation: (1) Framework specificity versus universal applicability; (2) Disclosure-focused standards versus lifecycle governance; and (3) Epistemic equivalence and the authority gradient. In the next sections we will detail each of these tensions.

#### 3.4.1 Tension 1: Framework specificity versus universal applicability.

The dominant approach to AI reporting governance has been to extend existing, design-specific reporting standards (CONSORT, TRIPOD, STARD, CLAIM) with AI-specific items. This produces instruments with strong methodological rigour within their target design but creates gaps for observational, implementation, and interdisciplinary AI studies. The Kolbinger et al.^(4)^ meta-analysis found that no single framework applied adequately across more than 60% of AI study types. Design-agnostic instruments, such as the KJA comprehensive checklist (Tier 3) and REFORMS (Tier 1), represent an alternative approach, trading methodological depth for breadth. Neither the specificity nor the universality strategy alone resolves the coverage problem; the field currently lacks an accepted hierarchical decision framework that tells researchers which instrument to apply when.

#### 3.4.2 Tension 2: Disclosure-focused standards versus lifecycle governance.

A substantial proportion of governance activity, particularly the Tier 4 editorial and institutional policies, focuses on authorship prohibition and disclosure at the point of manuscript submission. While necessary, these downstream controls address a narrow slice of AI governance. Upstream concerns, dataset diversity (STANDING Together), clinical implementation safety (DECIDE-AI), post-deployment monitoring, and environmental impact are addressed by a smaller set of instruments and receive less consistent enforcement. The heatmap in Fig 2 illustrates this asymmetry: authorship and disclosure coverage rates as primary in the generative AI, surgical, and editorial or policy groups (G3, G5, and G6), while post-deployment monitoring rates as primary only within the sociotechnical and lifecycle group (G2), and environmental sustainability receives only minor coverage across all six groups. This asymmetry may reflect the immediate pressure of responding to generative AI in editorial contexts, but it risks creating a governance architecture that is comprehensive at the point of publication and thin at the point of deployment.

#### 3.4.3 Tension 3: Epistemic equivalence and the authority gradient.

The governance literature treats instruments of very different evidential authority in similar rhetorical registers. A Tier 1 EQUATOR-listed guideline developed through multi-round Delphi with 50 + international experts (e.g., CONSORT-AI) and a Tier 3 preliminary checklist proposed by an editorial team (e.g., KJA) are frequently cited alongside each other without distinction. This risks what we term epistemic conflation: the appearance that the governance ecosystem is more robust than it is, because instrument count is treated as a proxy for governance quality. The evidence stratification introduced in this review is a step toward addressing this problem, but broader adoption of tier-sensitive language in the literature is needed.

## 4. Discussion

In this review, we contribute to the AI governance literature by mapping a broader segment of the governance ecosystem than previous reviews, within the boundaries of a PubMed and EQUATOR search, capturing 42 named frameworks across 100 included publications, including publications from 2025–2026 that were not available to prior syntheses. Additionally, it introduces an explicit evidence stratification, distinguishing Tier 1 EQUATOR-listed guidelines from Tier 3 emerging checklists, to address the epistemic conflation that characterizes much of the existing literature. It also articulates three governance tensions that help explain why framework proliferation has not translated into governance coherence.

The governance trajectory described, from model-centric reporting toward sociotechnical and generative AI oversight, mirrors developments in the broader AI ethics literature but with a distinctive publication-governance character. The rapid proliferation of Tier 4 editorial policies since 2023 is a response to a specific, urgent problem: the risk that AI-generated text, fabricated citations, and synthetic imagery undermine the verifiability of the published scientific record. Bibliometric analyses indicate that the vast majority of leading biomedical journals now explicitly prohibit AI authorship [10]. Suggesting this particular governance problem has achieved near-universal policy consensus. However, consensus on authorship prohibition has not been matched by consensus on disclosure standards. Significant variation persists in where disclosure should appear (acknowledgments versus methods versus cover letter), how much detail is required, and whether routine language assistance requires disclosure at all [11–13].

The unprecedented speed of governance proliferation since 2023 warrants explicit discussion in relation to the third governance tension (epistemic conflation and the authority gradient). Formal EQUATOR-registered guideline development typically requires 18–36 months of Delphi consensus activity, expert consultation, and iterative piloting before publication. The emergence of LLMs as mainstream tools from late 2022 onward created institutional pressure from editors, funders, and publishers to respond far faster than this infrastructure permits. The result is that the 2023–2026 governance phase is disproportionately populated by Tier 3 and Tier 4 instruments: editorial statements, preliminary checklists, and proposed frameworks that reflect institutional urgency rather than methodological maturity. Researchers and editors should interpret the growing count of governance instruments not as evidence of increasing methodological robustness, but as a signal that the field has not yet developed the coordination infrastructure needed to translate urgency into quality.

The deepest structural problem identified in this review is the gap between the governance ecosystem as it currently exists, characterized by design-specific checklists and editorial policies, and the governance ecosystem needed to address AI across the research lifecycle.Environmental impact and institutional accountability are addressed by only a small number of frameworks, and post-deployment monitoring and algorithmic equity receive substantive attention almost exclusively within the sociotechnical and lifecycle group (G2); none of these four concerns is the primary focus of a Tier 1 EQUATOR-listed guideline.DECIDE-AI partially addresses early-phase clinical deployment, and FUTURE-AI broadly addresses lifecycle governance, but neither provides the granular, enforcement-capable standards that high-stakes AI deployment in clinical settings requires. This gap is significant given the trajectory of AI adoption: as AI systems move from research tools to clinical infrastructure, point-of-publication governance will become insufficient.

The three governance tensions identified in this review, specificity versus applicability, disclosure versus lifecycle accountability, and epistemic conflation, collectively point toward the same policy implication: the field needs governance architecture rather than governance accumulation. Adding further isolated checklists is unlikely to resolve coverage gaps or reduce fragmentation; what is needed is a structured hierarchy that indicates which instrument applies at which stage of the research lifecycle, for which study design, and at which level of AI integration. The EQUATOR Network’s governance infrastructure provides a model for this kind of coordination, but its current focus on pre-publication reporting standards means it does not yet extend to deployment or post-publication governance.

### 4.1 Limitations

This review has methodological limitations that are worth noting. The search was restricted to PubMed and EQUATOR; Scopus, Web of Science, and broader grey literature were not searched, which may have led to under-representation of institutional guidance and non-English language instruments. The relevance-scoring algorithm used for pre-screening was not formally validated against an independent reference set, and its sensitivity and false-negative rate below the threshold were not empirically estimated; relevant sources scoring below the threshold may therefore have been missed. This limits the demonstrable completeness of the search, and the screening results should be interpreted with this uncertainty in mind. The Named EQUATOR search block retrieved over one million records due to common acronym terms; a retrieval cap was applied, potentially introducing selection bias within that block. Records scoring below the threshold may include relevant instruments and were not screened; this represents an inherent limitation of algorithmic pre-screening. Most included sources originate from North American and European institutions and are published in English, which may underrepresent guidance developed in other contexts. In addition, several Tier 3 instruments published in 2025–2026 reference PMIDs rather than fully indexed citations at the time of writing; readers should verify current publication status before use. Finally, because the search was restricted to PubMed and EQUATOR, and because the retrieved literature was predominantly English-language, this review should be read as a structured map of the indexed literature rather than as a comprehensive or globally representative account; in particular, it should not be read as characterizing governance activity in low- and middle-income countries or non-English publishing systems.

Individual publication-level exclusion reasons were not logged for all 788 records excluded during human screening. Because exclusion decisions were recorded at the category level, an accurate record-by-record exclusion table could not be reconstructed retrospectively. Although the predefined exclusion categories and aggregate screening counts are reported, this limits the reproducibility of individual exclusion decisions. Foundational organizational and technical AI standards, including ISO/IEC 42001 (AI Management System Standard) and the NIST Artificial Intelligence Risk Management Framework, were not identified by our search and did not meet the eligibility criteria for this review. These instruments are published by standards bodies rather than in biomedical journals and are not indexed in PubMed; furthermore, they address enterprise-level AI risk management rather than providing reporting guidance for the publication of AI research findings. Their relationship to publication-level governance warrants attention in future integrative reviews.

## 5. Conclusion

AI governance in healthcare publications has evolved from model performance checklists toward comprehensive sociotechnical and generative AI standards across four identifiable phases. Despite this growth, the governance ecosystem remains fragmented by evidence tier, domain coverage, and applicability. Three governance tensions, framework specificity versus universality, disclosure versus lifecycle accountability, and epistemic conflation, recur across framework groups and constrain progress toward coherent governance. Advancing the field requires evidence-tier-aware governance practice, harmonization of disclosure standards, and investment in governance architecture rather than further checklist proliferation.

## Supporting information

S1 Table Search string. (DOCX)

S2 Table Relevance scoring algorithm. (DOCX)

S3 Table PRISMA-style record counts. (DOCX)

S4 Table Characteristics of all included publications. (DOCX)

S5 Table Systematic search script. (DOCX)
