# Supplementary material for: Transparency, accountability, and scholarly integrity: A systematic narrative review of artificial intelligence governance frameworks in healthcare publication
Source: PLOS Digit Health. 2026 Oct 20;5(10):e0001783. doi: 10.1371/journal.pdig.0001783 (PMC13682662; doi:10.1371/journal.pdig.0001783)
Supplement: Supplementary file 1 [file pdig.0001783.s001.docx]

**Supporting Information — S1 File – Search String**

**Database:** PubMed (MEDLINE) via NCBI E-utilities API

**Supplementary source:** EQUATOR Network guideline library (equator-network.org) — manual search; 22 curated records added

**Search date:** 19 May 2026

**Date range covered: 1 January 2018 to 19 May 2026**

**Total records retrieved across all blocks:** 35,847 (before deduplication)

**Records after deduplication:** 30,276

Search strings were developed iteratively to maximise sensitivity across the governance landscape. MeSH headings were used where available and supplemented with free-text synonyms. All strings were executed with no language restriction. The search strategy was designed to capture formal EQUATOR-listed guidelines, peer-reviewed consensus frameworks, editorial policies, and emerging instruments. Full record counts per block are reported in S3 File.

Note on Block 3 (Named EQUATOR guidelines): several terms in this block (e.g., CLEAR, CHART, CLAIM, PROCESS, PRIME) are common acronyms with non-AI medical uses. This block retrieved over 1,000,000 raw records; a retrieval cap of 9,997 records was applied using PubMed's default relevance sort order. This cap is acknowledged as a potential source of selection bias within this block and is reported as a limitation in the main manuscript.

## Search Strings by Block

## Block 1: Core AI reporting

**Records retrieved:** 5,940

*Identifies publications addressing core AI/ML reporting standards in healthcare across any study design.*

### Boolean string (PubMed syntax):

("artificial intelligence"[MeSH] OR "machine learning"[MeSH] OR "deep learning"[tw] OR "neural network"[tw] OR "large language model*"[tw] OR "LLM"[tw] OR "generative AI"[tw] OR "foundation model*"[tw])

AND

("guideline*"[tw] OR "reporting standard*"[tw] OR "checklist*"[tw] OR "reporting item*"[tw] OR "reporting framework*"[tw] OR "consensus statement*"[tw] OR "Delphi"[tw] OR "EQUATOR"[tw])

AND

("healthcare"[tw] OR "health care"[tw] OR "clinical"[tw] OR "biomedical"[tw] OR "medicine"[tw] OR "nursing"[tw] OR "public health"[tw])

## Block 2: Publication ethics and AI

**Records retrieved:** 2,072

*Targets authorship, disclosure, peer review, and editorial policy in the context of AI use.*

### Boolean string (PubMed syntax):

("artificial intelligence"[MeSH] OR "machine learning"[MeSH] OR "generative AI"[tw] OR "large language model*"[tw] OR "ChatGPT"[tw] OR "GPT"[tw] OR "LLM"[tw])

AND

("authorship"[tw] OR "publication ethics"[tw] OR "research integrity"[tw] OR "peer review"[tw] OR "disclosure"[tw] OR "transparency"[tw] OR "editorial polic*"[tw] OR "journal polic*"[tw])

AND

("guideline*"[tw] OR "recommendation*"[tw] OR "framework*"[tw] OR "standard*"[tw] OR "policy"[tw])

## Block 3: Named EQUATOR guidelines

**Records retrieved:** 9,997 (capped)

*Directly retrieves publications citing known AI reporting guideline acronyms by name. Note: common acronyms (CLEAR, CHART, CLAIM) inflate retrieval; a cap of 9,997 records was applied.*

### Boolean string (PubMed syntax):

"CONSORT-AI"[tw] OR "SPIRIT-AI"[tw] OR "TRIPOD-AI"[tw] OR "TRIPOD+AI"[tw] OR "PROBAST-AI"[tw] OR "PROBAST+AI"[tw] OR "STARD-AI"[tw] OR "DECIDE-AI"[tw] OR "CLAIM"[tw] OR "FUTURE-AI"[tw] OR "STANDING Together"[tw] OR "GAMER"[tw] OR "CHART"[tw] OR "TRIPOD-LLM"[tw] OR "CANGARU"[tw] OR "MI-CLAIM"[tw] OR "CHEERS-AI"[tw] OR "REFORMS"[tw] OR "CLEAR"[tw] OR "METRICS"[tw] OR "CAIR"[tw] OR "GUIDE-AI"[tw] OR "AIHGIe"[tw] OR "TREG-AI"[tw] OR "QUADAS-AI"[tw] OR "R-AI-DIOLOGY"[tw] OR "PRIME"[tw] OR "STREAM-URO"[tw] OR "MINIMAR"[tw] OR "SCARE"[tw] OR "PROCESS"[tw] OR "STROCSS"[tw]

## Block 4: Systematic reviews of AI guidelines

**Records retrieved:** 411

*Identifies systematic reviews and meta-analyses evaluating AI reporting guidelines.*

### Boolean string (PubMed syntax):

("systematic review"[Publication Type] OR "scoping review"[tw] OR "meta-analysis"[Publication Type])

AND

("artificial intelligence"[MeSH] OR "machine learning"[MeSH])

AND

("reporting guideline*"[tw] OR "reporting standard*"[tw] OR "checklist*"[tw] OR "guideline quality"[tw] OR "AGREE"[tw])

## Block 5: Predictive model reporting

**Records retrieved:** 620

*Targets reporting of clinical prediction and prognostic models using AI/ML methods.*

### Boolean string (PubMed syntax):

("prediction model*"[tw] OR "prognostic model*"[tw] OR "diagnostic model*"[tw] OR "clinical prediction"[tw])

AND

("machine learning"[MeSH] OR "artificial intelligence"[MeSH] OR "deep learning"[tw])

AND

("reporting"[tw] OR "transparency"[tw] OR "checklist"[tw] OR "guideline"[tw])

## Block 6: LLM and chatbot reporting

**Records retrieved:** 6,221

*Captures publications addressing reporting of LLM-based studies, chatbot evaluations, and generative AI in clinical contexts.*

### Boolean string (PubMed syntax):

("large language model*"[tw] OR "ChatGPT"[tw] OR "GPT-4"[tw] OR "Bard"[tw] OR "Gemini"[tw] OR "Claude"[tw] OR "chatbot*"[tw])

AND

("reporting"[tw] OR "guideline*"[tw] OR "checklist*"[tw] OR "framework*"[tw] OR "standard*"[tw])

AND

("health"[tw] OR "medical"[tw] OR "clinical"[tw])

## Block 7: Radiology and medical imaging AI

**Records retrieved:** 5,339

*Targets AI reporting in radiology, radiomics, and medical imaging, capturing specialist checklists including CLAIM, CLEAR, and METRICS.*

### Boolean string (PubMed syntax):

("radiology"[MeSH] OR "radiomics"[tw] OR "medical imaging"[tw] OR "diagnostic imaging"[MeSH])

AND

("artificial intelligence"[MeSH] OR "deep learning"[tw] OR "machine learning"[MeSH])

AND

("reporting guideline*"[tw] OR "checklist*"[tw] OR "CLAIM"[tw] OR "CLEAR"[tw] OR "METRICS"[tw] OR "RQS"[tw] OR "STARD"[tw])

## Block 8: Specialty-specific AI guidelines

**Records retrieved:** 159

*Identifies specialty-adapted AI reporting frameworks across dermatology, cardiology, urology, surgery, ophthalmology, pathology, and oncology.*

### Boolean string (PubMed syntax):

("artificial intelligence"[MeSH] OR "machine learning"[MeSH])

AND

("dermatology"[tw] OR "cardiology"[tw] OR "urology"[tw] OR "surgery"[tw] OR "ophthalmology"[tw] OR "pathology"[tw] OR "oncology"[tw])

AND

("reporting guideline*"[tw] OR "checklist*"[tw] OR "reporting standard*"[tw])

## Block 9: Fairness, bias, and equity in AI reporting

**Records retrieved:** 1,953

*Addresses structural bias, algorithmic equity, and fairness-oriented reporting frameworks.*

### Boolean string (PubMed syntax):

("algorithmic bias"[tw] OR "fairness"[tw] OR "equity"[tw] OR "disparity"[tw] OR "representation"[tw])

AND

("artificial intelligence"[MeSH] OR "machine learning"[MeSH])

AND

("reporting"[tw] OR "guideline*"[tw] OR "framework*"[tw] OR "standard*"[tw] OR "checklist*"[tw])

AND

("health"[tw] OR "medical"[tw] OR "clinical"[tw])

## Block 10: Health economics and AI reporting

**Records retrieved:** 288

*Captures health economic evaluation reporting for AI-based interventions, including the CHEERS-AI framework.*

### Boolean string (PubMed syntax):

("cost-effectiveness"[tw] OR "health economic*"[tw] OR "economic evaluation"[tw] OR "CHEERS"[tw])

AND

("artificial intelligence"[MeSH] OR "machine learning"[MeSH])

AND

("reporting"[tw] OR "guideline*"[tw] OR "standard*"[tw])

## Block 11: Editorial and publisher policies

**Records retrieved:** 114

*Identifies editorial policies and publisher guidance from major organisations including ICMJE, WAME, and COPE.*

### Boolean string (PubMed syntax):

("ICMJE"[tw] OR "WAME"[tw] OR "COPE"[tw] OR "editorial board"[tw] OR "journal editor*"[tw] OR "publisher*"[tw])

AND

("artificial intelligence"[MeSH] OR "generative AI"[tw] OR "large language model*"[tw] OR "ChatGPT"[tw])

AND

("policy"[tw] OR "guideline*"[tw] OR "recommendation*"[tw] OR "disclosure"[tw] OR "authorship"[tw])

## Block 12: Real-world evidence and AI

**Records retrieved:** 1,111

*Captures AI reporting in real-world evidence and observational study contexts.*

### Boolean string (PubMed syntax):

("real-world evidence"[tw] OR "real world data"[tw] OR "observational stud*"[tw] OR "registry"[tw])

AND

("artificial intelligence"[MeSH] OR "machine learning"[MeSH] OR "deep learning"[tw])

AND

("reporting"[tw] OR "guideline*"[tw] OR "standard*"[tw] OR "framework*"[tw] OR "checklist*"[tw])

AND

("oncology"[tw] OR "cancer"[tw] OR "clinical"[tw] OR "healthcare"[tw])

## Block 13: Clustered and multi-centre prediction models

**Records retrieved:** 1,112

*Identifies reporting standards for prediction models in multi-centre, federated, and clustered data settings.*

### Boolean string (PubMed syntax):

("TRIPOD"[tw] OR "prediction model*"[tw] OR "prognostic model*"[tw])

AND

("cluster*"[tw] OR "multi-centre"[tw] OR "multicenter"[tw] OR "federated"[tw] OR "distributed"[tw] OR "external validation"[tw])

AND

("reporting"[tw] OR "guideline*"[tw] OR "checklist*"[tw] OR "transparency"[tw])

## Block 14: AI governance and regulation

**Records retrieved:** 488

*Captures governance, regulatory frameworks, and accountability instruments for AI in healthcare.*

### Boolean string (PubMed syntax):

("governance"[tw] OR "regulation"[tw] OR "regulatory framework"[tw] OR "AI Act"[tw] OR "FDA"[tw])

AND

("artificial intelligence"[MeSH] OR "machine learning"[MeSH])

AND

("health"[MeSH Subheading] OR "healthcare"[tw] OR "clinical"[tw])

AND

("reporting"[tw] OR "guideline*"[tw] OR "transparency"[tw] OR "accountability"[tw])

# EQUATOR Network Manual Search

# The EQUATOR Network guideline library was searched manually at equator-network.org/reporting-guidelines on 19 May 2026 using the terms 'artificial intelligence', 'machine learning', 'large language model', 'generative AI', and 'deep learning'. All identified reporting guidelines with AI or ML as a primary focus were extracted and added to the dataset as curated records (n = 22). These records were assigned a maximum relevance score (10) to ensure inclusion in the high-priority screening set.

# *EQUATOR = Enhancing the QUAlity and Transparency Of health Research; MeSH = Medical Subject Headings; [tw] = title/abstract text word field; [MeSH] = MeSH controlled vocabulary field.*
