# Supplementary material for: Transparency, accountability, and scholarly integrity: A systematic narrative review of artificial intelligence governance frameworks in healthcare publication
Source: PLOS Digit Health. 2026 Oct 20;5(10):e0001783. doi: 10.1371/journal.pdig.0001783 (PMC13682662; doi:10.1371/journal.pdig.0001783)
Supplement: Supplementary file 2 [file pdig.0001783.s002.docx]

**Supporting Information — S2 File - Relevance Scoring Algorithm**

*Documentation of pre-screening heuristic applied to PubMed records*

This file documents the relevance scoring algorithm applied to deduplicated PubMed records to prioritise records for human title and abstract screening. The algorithm is a pragmatic pre-screening heuristic, not a validated quality appraisal instrument. Its sole function was to reduce the screening burden from 30,276 unique records to a manageable high-priority subset while minimising the risk of excluding known frameworks.

**All final inclusion and exclusion decisions were made by human reviewers** applying the eligibility criteria defined in the main Methods section. The algorithm did not make inclusion decisions.

## Algorithm Logic

## Scoring formula

**score = Σ(inclusion signals matched) − 2 × Σ(exclusion signals matched)**

score = max(0, score) [floored at zero; negative scores not used] No upper score cap was applied (no maximum cap on total scores).

Scoring was performed by scanning the concatenated title and abstract text of each record using case-insensitive substring matching. Records with a final score ≥ 3 were designated high-priority and proceeded to full human title and abstract screening (n = 888; 2.9% of deduplicated records).

**Inclusion Signal Terms (n=16; each matched term +1 point)**

Each matched term contributes +1 point. All 16 terms are checked independently; multiple matches accumulate.

| **No.** | **Term (case-insensitive, substring match)** | **No.** | **Term** |
| --- | --- | --- | --- |
| 1 | reporting guideline | 9 | checklist |
| 2 | reporting checklist | 10 | appraisal tool |
| 3 | reporting standard | 11 | quality assessment |
| 4 | delphi | 12 | systematic review |
| 5 | publication ethics | 13 | authorship |
| 6 | consensus | 14 | disclosure |
| 7 | equator | 15 | transparency |
| 8 | framework | 16 | editorial policy |

**Named Guideline Acronym Bonus (n=19 acronyms used in the implemented script; +2 points per record, maximum one bonus per record)**

If any of the following acronyms are found in the record title (case-insensitive), an additional +2 points are added. Maximum one bonus per record.

The following 19 acronyms were used in the implemented script (S5 File compute_relevance_score function): consort-ai, spirit-ai, tripod, probast, stard-ai, decide-ai, claim, future-ai, gamer, chart, cair, reforms, cheers-ai, clear, metrics, minimar, quadas, standing together, guide-ai — as implemented in S5 File.

**Exclusion Signal Terms (n=15; 15 exclusion signal terms; each matched term −2 points)**

Each matched term contributes −2 points. These terms indicate primary clinical studies using AI as an intervention rather than governance or reporting guidance.

| **No.** | **Term** | **No.** | **Term** |
| --- | --- | --- | --- |
| 1 | randomized controlled trial | 8 | case report |
| 2 | randomised controlled trial | 9 | case series |
| 3 | phase ii | 10 | animal model |
| 4 | phase iii | 11 | murine |
| 5 | drug trial | 12 | mouse model |
| 6 | vaccine | 13 | in vitro |
| 7 | surgery outcome | 14 | in vivo |
| 15 | cell line |  |  |

**EQUATOR Curated Records**

The 22 manually curated Records identified through the EQUATOR Network manual search (n=22) were combined with PubMed records, deduplicated, and scored using the same relevance-scoring function as all other records. This ensured that all known EQUATOR-registered AI reporting guidelines were captured irrespective of their title/abstract keyword profile.

**Score Distribution**

| **Score** | **Records (n)** | **Proportion (%)** |
| --- | --- | --- |
| 3 | ~310 | 34.9 |
| 4 | ~245 | 27.6 |
| 5 | ~178 | 20.0 |
| 6 | ~98 | 11.0 |
| 7+ | ~57 | 6.4 |
| **Total** | **888** | **100.0** |

**Threshold Selection and Validation**

The threshold of ≥ 3 was selected by verifying that all 34 frameworks identified in prior narrative work by the authors were present in the high-priority set. All 34 known frameworks scored ≥ 4. No formal external validation against a gold standard was performed.

**Limitations**

**Unscreened records:** Records scoring < 3 were not screened. Important governance documents with low keyword density in titles and abstracts (e.g., regulatory white papers, institutional policy statements) may have been missed. This is acknowledged as a limitation in the main manuscript.

**Block 3 retrieval cap:** The Named EQUATOR search block (Block 3) retrieved over 1,000,000 raw records due to common acronyms used as free-text terms. A cap of 9,997 records was applied using PubMed's default sort order, potentially introducing selection bias toward recently indexed records within this block.

**No inter-rater reliability:** Threshold selection was not subject to formal inter-rater reliability calculation.

**English language only:** The algorithm operates on English title and abstract text. Non-English records without English abstracts received a default score of 0.

**Reproducibility**

The algorithm is fully implemented in S5 File (ai_guidelines_systematic_search_v2.py) within the compute_relevance_score() function and can be re-run against any PubMed dataset. Output files include the scored deduplicated dataset with relevance_score and relevance_signals columns visible for audit.
