# Supplementary material for: Transparency, accountability, and scholarly integrity: A systematic narrative review of artificial intelligence governance frameworks in healthcare publication
Source: PLOS Digit Health. 2026 Oct 20;5(10):e0001783. doi: 10.1371/journal.pdig.0001783 (PMC13682662; doi:10.1371/journal.pdig.0001783)
Supplement: Supplementary file 3 [file pdig.0001783.s003.docx]

**Supporting Information — S3 File - PRISMA-Style Record Counts**

**Search Information**

**Databases:** PubMed (MEDLINE) via NCBI E-utilities; EQUATOR Network (manual)

**Search date:** May 2026 (update on May 19)

**Date range:** 1 January 2018 to 19 May 2026

**Number of search blocks:** 14 (PubMed) + 1 (EQUATOR manual)

**Full search strings:** See S1 File

**Algorithm documentation:** See S2 File

**Records Identified per Search Block**

| **Block** | **Search block label** | **Records retrieved (n)** |
| --- | --- | --- |
| 1 | Core AI reporting | **5,940** |
| 2 | Publication ethics and AI | **2,072** |
| 3 | Named EQUATOR guidelines (capped at 9,997) | **9,997** |
| 4 | Systematic reviews of AI guidelines | **411** |
| 5 | Predictive model reporting | **620** |
| 6 | LLM and chatbot reporting | **6,221** |
| 7 | Radiology and medical imaging AI | **5,339** |
| 8 | Specialty-specific AI guidelines | **159** |
| 9 | Fairness, bias, and equity in AI reporting | **1,953** |
| 10 | Health economics and AI | **288** |
| 11 | Editorial and publisher policies | **114** |
| 12 | Real-world evidence and AI | **1,111** |
| 13 | Clustered and multi-centre prediction models | **1,112** |
| 14 | AI governance and regulation | **488** |
| — | EQUATOR Network (manual curation) | **22** |
|  | **TOTAL — all blocks combined (before deduplication)** | **35,847** |

**Deduplication and Screening**

| **Stage** | **Records (n)** |
| --- | --- |
| Total records identified (all blocks combined) | 35,847 |
| Duplicates removed (matched by PMID, DOI, and normalised title) | 5,571 |
| **Records after deduplication** | **30,276** |
| Records with relevance score ≥ 3 (algorithm pre-screening; see S2 File) | 888 |
| Records excluded at title/abstract screening (ineligible) | 788 |
| Records proceeding to full eligibility assessment | 100 |
| Records excluded at full eligibility assessment | 0 |
| **Sources included in final synthesis** | **100** |

**Notes**

**Block 3 retrieval cap:** Block 3 (Named EQUATOR guidelines) queried common acronyms as free-text terms (e.g., CLEAR, CHART, CLAIM, PROCESS). The raw retrieval exceeded 1,000,000 records; a cap of 9,997 was applied using PubMed's default sort. This is acknowledged as a potential source of selection bias and is reported as a limitation in the main manuscript.

**Relevance scoring:** The algorithm used to generate the 888 high-priority records is fully documented in S2 File. Records scoring < 3 were not screened; this represents an inherent limitation of the pre-screening approach. Of the 30,276 deduplicated records, 29,388 scored below the threshold and did not proceed to human screening; 888 scored ≥3 and proceeded to human screening. Individual records not advanced to human screening were not assessed at the document level; this represents an inherent limitation of algorithmic pre-screening, acknowledged in the manuscript Limitations section.

**EQUATOR curation: 22 records from the EQUATOR Network manual search were included in the 888 high-priority records.**

**PRISMA 2020 alignment:** These counts correspond to the PRISMA 2020 flow diagram (Fig 1) in the main manuscript. Full PRISMA 2020 checklist is available from the corresponding author on request.
