# Supplementary material for: Transparency, accountability, and scholarly integrity: A systematic narrative review of artificial intelligence governance frameworks in healthcare publication
Source: PLOS Digit Health. 2026 Oct 20;5(10):e0001783. doi: 10.1371/journal.pdig.0001783 (PMC13682662; doi:10.1371/journal.pdig.0001783)
Supplement: Supplementary file 4 [file pdig.0001783.s004.docx]

**S4 File — Characteristics of All 100 Included Publications**

| Group | APA Citation | Year | Publication Type | Tier / Category | Key Aim / Focus | Target Context | EQUATOR Listed |
| --- | --- | --- | --- | --- | --- | --- | --- |
| Group 1 — Predictive and Diagnostic AI Frameworks (n=11; 10 governance instruments [9 Tier 1, 1 Tier 3] + 1 contextual evidence source) | | | | | | | |
| G1 | Liu X, Cruz Rivera S, Moher D, et al. (2020). Reporting guidelines for clinical trial reports for interventions involving artificial intelligence: The CONSORT-AI Extension. BMJ, 370, m3164. | 2020 | Reporting Guideline | **Tier 1** | Standardises reporting of RCTs evaluating AI interventions; Delphi-developed | Randomised controlled trials of AI interventions | Yes |
| G1 | Cruz Rivera S, Liu X, Chan AW, et al. (2020). Guidelines for clinical trial protocols for interventions involving AI: The SPIRIT-AI Extension. Lancet Digital Health, 2(10), e549–e560. | 2020 | Reporting Guideline | **Tier 1** | Extends SPIRIT to standardise AI clinical trial protocols | Clinical trial protocol development | Yes |
| G1 | Collins GS, Moons KGM, Dhiman P, et al. (2024). TRIPOD+AI statement: Updated guidance for reporting clinical prediction models using regression or machine learning. BMJ, 385, e078378. | 2024 | Reporting Guideline | **Tier 1** | Updated TRIPOD for ML prediction model reporting; supersedes 2015 TRIPOD | Prediction model development and external validation | Yes |
| G1 | Moons KGM, et al. (2025). PROBAST+AI: An updated quality, risk of bias, and applicability assessment tool for prediction model studies using AI methods. BMJ, 388, e082505. | 2025 | Appraisal Tool | **Tier 1** | Updated risk-of-bias tool for AI/ML prediction models; paired with TRIPOD+AI | Systematic reviews and critical appraisal of prediction models | Yes |
| G1 | Sounderajah V, Guni A, Liu X, et al. (2025). The STARD-AI reporting guideline for diagnostic accuracy studies using artificial intelligence. Nature Medicine, 31(10), 3283–3289. | 2025 | Reporting Guideline | **Tier 1** | Final STARD-AI guideline; extends STARD 2015 to AI-based diagnostic accuracy studies; modified Delphi; EQUATOR-listed | Diagnostic test accuracy studies using AI | Yes |
| G1 | Mongan J, Moy L, Kahn CE Jr. (2020). Checklist for Artificial Intelligence in Medical Imaging (CLAIM). Radiology: Artificial Intelligence, 2(2), e200029. | 2020 | Reporting Guideline | **Tier 1** | Best practice checklist for AI in medical imaging research | Medical imaging AI research | Yes |
| G1 | Tejani AS, et al. (2024). Checklist for Artificial Intelligence in Medical Imaging (CLAIM): 2024 Update. Radiology: Artificial Intelligence, 6, e240300. | 2024 | Reporting Guideline | **Tier 1** | Updates CLAIM for current medical imaging AI; same instrument, updated publication | Medical imaging AI — updated guidance | Yes |
| G1 | Guni A, Sounderajah V, Whiting P, et al. (2024). QUADAS-AI protocol. JMIR Research Protocols. | 2024 | Appraisal Tool | **Tier 3** | Protocol for QUADAS-AI risk-of-bias tool adapted for AI diagnostic accuracy studies | Systematic reviews of AI diagnostic accuracy studies | No |
| G1 | Elvidge J, Hawksworth C, Avsar TS, et al. (2024). Consolidated Health Economic Evaluation Reporting Standards for Interventions That Use Artificial Intelligence: CHEERS-AI. Value in Health. | 2024 | Reporting Guideline | **Tier 1** | First EQUATOR-listed reporting standard for health economic evaluations of AI interventions | Health economic evaluation of AI-based interventions | Yes |
| G1 | Norgeot B, et al. (2020). Minimum information about clinical artificial intelligence modeling: The MI-CLAIM checklist. Nature Medicine. | 2020 | Reporting Guideline | **Tier 1** | Minimum information checklist for clinical AI modelling; EQUATOR-listed | Clinical AI model development and reporting | Yes |
| G1 | Shelmerdine SC, et al. (2021). Review of study reporting guidelines for clinical studies using artificial intelligence in healthcare. BMJ Health and Care Informatics. | 2021 | Systematic Review | Contextual | Reviews STARD-AI, TRIPOD-AI, CONSORT-AI; contextual evidence supporting narrative synthesis | Overview of AI-specific EQUATOR reporting extensions | — |
| Group 2 — Sociotechnical and Lifecycle Governance (n=20; 8 governance instruments [2 Tier 1, 3 Tier 2, 2 Tier 3, 1 Tier 4] + 12 contextual evidence sources) | | | | | | | |
| G2 | Vasey B, Nagendran M, Campbell B, et al. (2022). Reporting guideline for the early-stage clinical evaluation of decision support systems driven by artificial intelligence: DECIDE-AI. Nature Medicine, 28, 924–933. | 2022 | Reporting Guideline | **Tier 1** | Primary publication of DECIDE-AI; Delphi-developed; EQUATOR-listed | Early-stage clinical evaluation of AI decision support systems | Yes |
| G2 | Vasey B, et al. (2022). Reporting guideline for the early-stage clinical evaluation of decision support systems driven by artificial intelligence: DECIDE-AI. BMJ. | 2022 | Reporting Guideline | **Tier 1** | Parallel simultaneous BMJ publication of the DECIDE-AI guideline; same Delphi process and EQUATOR registration as Nature Medicine paper; two publications, one named instrument | Early-stage AI clinical evaluation — simultaneous BMJ publication | Yes |
| G2 | Lekadir K, et al. (2025). FUTURE-AI: International consensus guideline for trustworthy and deployable artificial intelligence in healthcare. BMJ, 388, e081554. | 2025 | Consensus Guideline | **Tier 2** | Covers full AI lifecycle: fairness, universality, traceability, usability, robustness, explainability | Entire healthcare AI lifecycle governance | No |
| G2 | Alderman JE, Palmer J, Laws E, et al. (2023). Tackling algorithmic bias and promoting transparency in health datasets: The STANDING Together consensus recommendations. Lancet Digital Health. [Updated 2025] | 2023 | Consensus Standard | **Tier 2** | Addresses root cause of algorithmic bias through transparency in health dataset curation | Health dataset diversity and training data governance | No |
| G2 | Ministry of Health & Health Sciences Authority, Singapore. (2026). Artificial intelligence in healthcare guidelines (AIHGIe 2.0). | 2026 | Governance Framework | **Tier 4** | Standardises transparent AI development and reporting in healthcare from a regulatory perspective | AI development and deployment governance (Singapore context) | No |
| G2 | Liu N, et al. (2023). Generative artificial intelligence in healthcare: Ethical considerations and assessment checklist (TREG-AI). eClinicalMedicine. | 2023 | Proposed Framework | **Tier 3** | Proposes TREG-AI checklist for ethical principles in generative AI research | Ethical reporting of generative AI in healthcare research | No |
| G2 | Avram R, et al. (2024). A responsible framework for applying artificial intelligence on medical images and signals at the point of care. | 2024 | Framework Proposal | **Tier 3** | Responsible framework for applying AI on medical images at the clinical point of care | Point-of-care AI implementation governance | No |
| G2 | Vollmer S, et al. (2020). Machine learning and AI research for patient benefit: 20 critical questions on transparency, reproducibility, ethics and effectiveness. BMJ. | 2020 | Expert Framework | **Tier 2** | 20 TREE critical questions for evaluating AI for patient benefit; multi-author expert consensus published in BMJ | Patient benefit-oriented AI evaluation framework | No |
| G2 | Crossnohere NL, Elsaid M, Paskett J, et al. (2022). Guidelines for artificial intelligence in medicine: Literature review and content analysis of frameworks. Journal of Medical Internet Research, 24(8), e36823. | 2022 | Scoping Review | Contextual | Content analysis of existing AI reporting frameworks; contextual evidence supporting narrative synthesis | Overview and mapping of AI reporting frameworks | — |
| G2 | Wang Y, Li N, Chen L, et al. (2023). Guidelines, consensus statements, and standards for the use of artificial intelligence in medicine: Systematic review. Journal of Medical Internet Research. | 2023 | Systematic Review | Contextual | Analyses methodological quality of AI reporting guidelines using AGREE II; contextual evidence | AI guideline development quality assessment | — |
| G2 | Kolbinger FR, Veldhuizen GP, Zhu J, et al. (2024). Reporting guidelines in medical artificial intelligence: A systematic review and meta-analysis. Communications Medicine, 4, 71. | 2024 | Systematic Review / Meta-analysis | Contextual | Meta-analysis of 26 reporting guidelines; contextual evidence supporting narrative synthesis | Coverage and overlap analysis of medical AI reporting guidelines | — |
| G2 | Alnaimat F, et al. (2025). Guidelines and standard frameworks for artificial intelligence in medicine: A systematic review. JAMIA Open, 8(1), ooae155. | 2025 | Systematic Review | Contextual | AGREE II evaluation identifying variability across frameworks; contextual evidence | AI guideline quality assessment and applicability | — |
| G2 | Zrubka Z, Kovacs L, Nezhad HM, et al. (2025). Artificial intelligence in medicine: A systematic review of guidelines for the reporting and interpretation of studies. Acta Polytechnica Hungarica. | 2025 | Systematic Review | Contextual | Reviews AI reporting guidelines; notes checklist fatigue; contextual evidence | Systematic evaluation of AI reporting guidelines | — |
| G2 | Ibrahim H, Liu X, Denniston A. (2021). Reporting guidelines for artificial intelligence in healthcare research. Clinical and Experimental Ophthalmology. | 2021 | Review | Contextual | Highlights the need for AI-specific EQUATOR reporting extensions; contextual evidence | AI reporting guidelines — narrative overview | — |
| G2 | Teo ZL, Kwee A, Lim JCW, et al. (2023). Artificial intelligence innovation in healthcare: Relevance of reporting guidelines for clinical translation. Annals of the Academy of Medicine, Singapore. | 2023 | Review | Contextual | Framework for utilising reporting guidelines to standardise AI clinical translation; contextual evidence | AI clinical translation and reporting alignment | — |
| G2 | Collins GS. (2024). Making the black box more transparent: Improving the reporting of AI studies in healthcare. BMJ. | 2024 | Editorial Commentary | Contextual | Expert editorial discussing TRIPOD+AI for improving AI study reporting; contextual evidence | AI reporting transparency — expert commentary | — |
| G2 | Alryalat SA, et al. (2024). Systematic review of reporting guidelines for large language models in healthcare research. | 2024 | Systematic Review | Contextual | Assesses reporting guidelines for LLM research; contextual evidence supporting narrative synthesis | LLM-specific reporting guidance | — |
| G2 | Huo B, Collins GS, Cacciamani GE, et al. (2025). Reporting guidelines for studies involving generative AI: What do I use, and when? npj Digital Medicine. | 2025 | Review | Contextual | Decision framework for selecting among competing AI reporting guidelines; contextual evidence — distinct from the CANGARU guideline-development protocol | AI guideline navigation and selection | — |
| G2 | Shiferaw KB, et al. (2024). Calibrating CONSORT-AI with FAIR principles to enhance reproducibility in AI-driven clinical trials. | 2024 | Methodological Paper | Contextual | Integrates CONSORT-AI with FAIR data principles; contextual evidence supporting narrative synthesis | AI reproducibility and FAIR data alignment | — |
| G2 | Campbell JR, et al. (2020). Reporting guidelines for artificial intelligence in medical research. Ophthalmology. | 2020 | Review | Contextual | References EQUATOR reporting guidelines for transparency; contextual evidence | AI reporting transparency in ophthalmology | — |
| Group 3 — Generative AI and LLM-Specific Standards (n=13; 11 governance instruments [3 Tier 1, 2 Tier 2, 6 Tier 3] + 2 contextual evidence sources) | | | | | | | |
| G3 | Luo X, et al. (2025). Reporting guideline for the use of Generative Artificial Intelligence in medical research (GAMER). BMJ Evidence-Based Medicine, 30, 390–400. | 2025 | Reporting Guideline | **Tier 1** | First international consensus GenAI reporting guideline; EQUATOR-listed; international Delphi | Generative AI use in medical research and manuscript preparation | Yes |
| G3 | The CHART Collaborative. (2025). Reporting guideline for chatbot health advice studies: The Chatbot Assessment Reporting Tool (CHART). JAMA Network Open; Artif Intell Med; Ann Fam Med; Br J Surg. | 2025 | Reporting Guideline | **Tier 1** | Multi-journal endorsed guideline for chatbot health advice evaluation; EQUATOR-listed | Chatbot health advice evaluation and reporting | Yes |
| G3 | Gallifant J, Afshar M, Ameen S, et al. (2025). The TRIPOD-LLM reporting guideline for studies using large language models. Nature Medicine, 31, 60–69. | 2025 | Reporting Guideline | **Tier 1** | Extends TRIPOD to LLM development and evaluation; international expert consensus; EQUATOR-listed | LLM development and evaluation reporting | Yes |
| G3 | Cacciamani GE, Eppler MB, Ganjavi C, et al. (2023). Development of the ChatGPT, Generative Artificial Intelligence and Natural Large Language Models for Accountable Reporting and Use (CANGARU) Guidelines. arXiv:2307.08974. | 2023 | Development Protocol | **Tier 3** | CANGARU guideline-development protocol; EQUATOR lists as under development at search date; preliminary instrument pending finalisation | Generative AI and LLM use in research and publication | Under development |
| G3 | Hryciw BN, et al. (2023). Guiding principles and proposed classification for responsible adoption of AI in scientific writing in medicine. Frontiers in Artificial Intelligence. | 2023 | Framework Proposal | **Tier 3** | Proposed classification system for adopting AI in medical scientific writing; single team; no Delphi | AI in scientific writing — proposed ethical classification | — |
| G3 | Mese I, Akinci D'Antonoli T, Bluethgen C, et al. (2026). Reporting checklist for foundation and large language models in medical research (REFINE): An international consensus guideline. Diagnostic and Interventional Radiology. PMID:41742713. | 2026 | Reporting Checklist | **Tier 2** | International consensus checklist for foundation models and LLMs; modified Delphi (54 panelists); not EQUATOR-listed at search date | Foundation model and LLM reporting in medicine | No |
| G3 | Khoruzhaya AN, Varyukhina MD, Erizhokov RA, et al. (2026). MEDAI-LLM-SUMM: A reporting checklist for medical text summarization studies using large language models. Frontiers in Digital Health, 8, 1761601. PMID:41847286. | 2026 | Reporting Checklist | **Tier 2** | First reporting checklist targeting LLM-based medical text summarisation; three-round multidisciplinary expert consensus; not EQUATOR-listed | LLM text summarisation research | No |
| G3 | Park SH, Suh CH, Lee JH, et al. (2025). Minimum reporting items for clear evaluation of accuracy reports of large language models in healthcare (MI-CLEAR-LLM): 2025 updates. Korean Journal of Radiology, 26(12), 1123–1132. PMID:41199132. | 2025 | Reporting Checklist | **Tier 3** | Minimum reporting items for LLM accuracy studies in healthcare; preliminary; pending endorsement | LLM accuracy reporting in healthcare | No |
| G3 | Gottlieb M, et al. (2025). PRISMA-trAIce: Transparent reporting of AI in systematic literature reviews. JMIR AI. PMID:41370833. | 2025 | Reporting Guideline | **Tier 3** | Extends PRISMA to systematic reviews using AI assistance; protocol stage; not yet EQUATOR-registered | AI-assisted systematic review reporting | No |
| G3 | Fehring L, Frings J, Rust P, et al. (2025). Extension of the Consolidated Criteria for Reporting Qualitative Research guideline to large language models (COREQ+LLM): Protocol for a multiphase study. JMIR Research Protocols, 14, e78682. PMID:40991937. | 2025 | Reporting Guideline | **Tier 3** | Adapts COREQ qualitative reporting to LLM-mediated data collection; preliminary; protocol-stage | LLM-mediated qualitative research | No |
| G3 | Mehta N, Felder RM, Kodish E, et al. (2026). A call for clarity: A unified checklist for reporting use of large language models in writing scientific manuscripts. Research Integrity and Peer Review, 11, 24. PMID:42106879. | 2026 | Proposed Checklist | **Tier 3** | Proposes harmonised LLM reporting standards; preliminary; no documented multi-stakeholder consensus | LLM use in scientific writing | No |
| G3 | Park SH, et al. (2023). Reporting guidelines for artificial intelligence studies in healthcare. Korean Journal of Radiology. | 2023 | Review | Contextual | Reviews AI reporting guidelines including LLM prompt documentation; contextual evidence | AI reporting — LLM prompt documentation | — |
| G3 | Park SH, et al. (2024). Reporting guidelines for AI studies in healthcare: What is new in 2024. Korean Journal of Radiology. | 2024 | Review | Contextual | Updates on reporting guidelines including LLM-specific frameworks; contextual evidence supporting narrative synthesis | AI reporting updates and LLM frameworks | — |
| Group 4 — Specialty-Specific AI Frameworks (n=11; 10 governance instruments [2 Tier 1, 7 Tier 2, 1 Tier 3] + 1 contextual evidence source) | | | | | | | |
| G4 | Sengupta PP, et al. (2020). PRIME: Proposed requirements for cardiovascular imaging-related machine learning evaluation. JACC: Cardiovascular Imaging. | 2020 | Reporting Guideline | **Tier 2** | Domain-specific ML evaluation framework for cardiovascular imaging; expert consensus; EQUATOR-listed | Cardiovascular imaging AI reporting | Yes |
| G4 | Kagiyama N, Tokodi M, Hathaway QA, et al. (2026). PRIME 2.0: Proposed requirements for cardiovascular imaging-related multimodal-AI evaluation. JACC: Cardiovascular Imaging, 19(2), 225–251. PMID:40892627. | 2026 | Reporting Guideline | **Tier 2** | Updates PRIME for multimodal AI; expert consensus; EQUATOR-listed | Cardiovascular multimodal AI reporting | Yes |
| G4 | Kwong JCC, et al. (2021). STREAM-URO: STandardized REporting of Machine learning Applications in Urology. European Urology Focus. | 2021 | Reporting Guideline | **Tier 2** | Standardised ML reporting for urology; specialty expert consensus; EQUATOR-listed | ML reporting in urology | Yes |
| G4 | Haller S, et al. (2022). R-AI-DIOLOGY: Recommendations for use of AI in clinical neuroradiology. Neuroradiology. | 2022 | Appraisal Tool | **Tier 2** | Practical appraisal tool for neuroradiologists evaluating commercial AI; expert recommendations | Neuroradiology AI appraisal | No |
| G4 | Daneshjou R, et al. (2022). CLEAR Derm: Transparent reporting for image-based AI in dermatology. JAMA Dermatology. | 2022 | Reporting Guideline | **Tier 2** | Addresses dermatological imaging biases; expert consensus; not EQUATOR-listed | Dermatology AI image-based reporting | No |
| G4 | Kocak B, et al. (2023). CLEAR: CheckList for EvaluAtion of Radiomics Research. Insights into Imaging. | 2023 | Reporting Guideline | **Tier 1** | Step-by-step radiomics reporting; ESR/EuSoMII endorsed; EQUATOR-listed. Distinct from CLEAR Derm. | Radiomics research evaluation and reporting | Yes |
| G4 | Kocak B, et al. (2024). METRICS: METhodological RadiomICs Score. Insights into Imaging. | 2024 | Appraisal Tool | **Tier 1** | 30-item radiomics quality scoring; EuSoMII-endorsed; EQUATOR-listed. Distinct from METRICS GenAI. | Radiomics study quality assessment | Yes |
| G4 | Olczak J, Pavlopoulos J, Prijs J, et al. (2021). Presenting artificial intelligence, deep learning, and machine learning studies to clinicians and healthcare stakeholders: An introductory reference with a guideline and a Clinical AI Research (CAIR) checklist proposal. Acta Orthopaedica, 92(5), 513–525. | 2021 | Reporting Guideline | **Tier 3** | General-purpose clinical AI research appraisal checklist; described by its authors as a proposal; EQUATOR-listed | Clinical AI research appraisal across specialties | Yes |
| G4 | Zhang X, Ferry J, Hewson DW, et al. (2025). Guidance for reporting artificial intelligence technology evaluations for ultrasound scanning in regional anaesthesia (GRAITE-USRA): An international multidisciplinary consensus reporting framework. Anaesthesia, 80, 1528–1539. PMID:40963489. | 2025 | Reporting Guidance | **Tier 2** | Specialty-specific guidance for AI in ultrasound-guided regional anaesthesia; international modified Delphi (67 experts) following EQUATOR development methodology; not EQUATOR-listed | Ultrasound AI reporting | No |
| G4 | Kottlors J, Iuga AI, Bluethgen C, et al. (2026). Guidelines for reporting studies on large language models in radiology: An international Delphi expert survey (FLAIR). Radiology, 318(2), e250913. PMID:41631991. | 2026 | Consensus Guideline | **Tier 2** | International Delphi for radiology LLM reporting standards; not EQUATOR-listed at search date | LLM reporting in radiology — specialty focus | No |
| G4 | Piotrowski T, et al. (2021). Ethical issues in AI radiology: How are they reported in research articles? Journal of Medical Science, 90(2), e513. | 2021 | Review | Contextual | Evaluates ethical reporting in radiology AI using STARD criteria; contextual evidence | Ethical reporting in radiology AI research | — |
| Group 5 — Surgical and Procedural Reporting Updates (n=3; 3 governance instruments [3 Tier 1] + 0 contextual evidence sources) | | | | | | | |
| G5 | Kerwan A, Al-Jabir A, Mathew G, et al. (2025). Revised Surgical CAse REport (SCARE) Guideline: An update for the age of artificial intelligence. Premier Journal of Science. | 2025 | Reporting Guideline | **Tier 1** | Integrates AI-specific domain into SCARE; consortium consensus; EQUATOR-listed update | Surgical case reports with AI | Yes |
| G5 | Agha RA, Mathew G, Rashid R, et al. (2025). Revised Preferred Reporting of Case Series in Surgery (PROCESS) Guideline: An update for the age of artificial intelligence. Premier Journal of Science. | 2025 | Reporting Guideline | **Tier 1** | Updates PROCESS with AI domain; consortium consensus; EQUATOR-listed update | Surgical case series with AI | Yes |
| G5 | Agha RA, et al. (2025). Revised Strengthening the Reporting of Cohort, Cross-Sectional and Case-Control Studies in Surgery (STROCSS) 2025. Premier Journal of Science. | 2025 | Reporting Guideline | **Tier 1** | Updates STROCSS 2025 with AI domain; consortium consensus; EQUATOR-listed update | Surgical cohort and observational studies with AI | Yes |
| Group 6 — Editorial and Publication Policy (n=42; 27 governance instruments [1 Tier 1, 2 Tier 3, 24 Tier 4] + 15 contextual evidence sources) | | | | | | | |
| G6 | International Committee of Medical Journal Editors. (2024). Recommendations for the conduct, reporting, editing, and publication of scholarly work in medical journals. | 2024 | Editorial Policy | **Tier 4** | Mandates detailed AI use description; prohibits AI-generated citations; bans AI authorship | AI authorship and disclosure in biomedical journals | — |
| G6 | Zielinski C, Winker MA, Aggarwal R, et al. (2023). Chatbots, generative AI, and scholarly manuscripts: WAME recommendations. WAME. | 2023 | Editorial Policy | **Tier 4** | Prohibits chatbot authorship; assigns legal and ethical responsibility to human authors | AI authorship policy for journals and editors | — |
| G6 | COPE Council. (2021/2023). Authorship and AI tools: COPE position statement. | 2023 | Policy Statement | **Tier 4** | Establishes ethical principles barring AI authorship; governs editorial AI decision-making | AI authorship ethics for publishers and editors | — |
| G6 | National Institutes of Health. (2023). Prohibition on AI in NIH peer review. Notice NOT-OD-23-149. | 2023 | Institutional Policy | **Tier 4** | Federal prohibition on AI use in NIH peer review to maintain proposal confidentiality | AI in grant peer review — federal policy | — |
| G6 | National Institutes of Health. (2025). Supporting fairness and originality in NIH research applications. Notice NOT-OD-25-132. | 2025 | Institutional Policy | **Tier 4** | Limits and fairness directives for AI use in NIH grant applications | AI use in research grant applications | — |
| G6 | European Research Council. (2026). Use of AI in grant proposal evaluation. | 2026 | Institutional Policy | **Tier 4** | Restricts reviewer AI use due to confidentiality and integrity concerns | AI in ERC grant evaluation | — |
| G6 | World Health Organization. (2024). Ethics and governance of artificial intelligence for health: Guidance on large multi-modal models. WHO. | 2024 | Global Guidance | **Tier 4** | Sets global ethical baseline for deploying generative models in human health settings | Global AI ethics and governance for health | — |
| G6 | European Commission. (2024). Living guidelines on the responsible use of generative AI in research. | 2024 | Regulatory Guidance | **Tier 4** | Consolidates European guidelines on transparent use and disclosure of generative AI in research | Generative AI in European research governance | — |
| G6 | Harvard Medical School. (2024). HMS recommendations and approved tools for responsible generative AI use. | 2024 | Institutional Guideline | **Tier 4** | Institutional guidance for secure AI use with sensitive data | Institutional AI governance — academic medical centre | — |
| G6 | Hernandez-Boussard T, et al. (2020). MINIMAR: Minimum information for medical AI reporting. Journal of the American Medical Informatics Association. | 2020 | Proposed Framework | **Tier 3** | Proposes minimum information criteria for medical AI reporting; no Delphi; EQUATOR-listed | Minimum AI reporting information framework | Yes |
| G6 | Kwak SG, Kim J. (2025). Comprehensive reporting guidelines and checklist for studies developing and utilizing AI models. Korean Journal of Anesthesiology. | 2025 | Reporting Guideline | **Tier 3** | Universal design-agnostic checklist; single journal; two authors; no multi-stakeholder Delphi | Universal AI reporting checklist (design-agnostic) | No |
| G6 | Kapoor S, Cantrell EM, Peng K, et al. (2024). REFORMS: Consensus-based recommendations for ML-based science. Science Advances. doi:10.1126/sciadv.adk3452. | 2024 | Consensus Checklist | **Tier 1** | 32-item consensus checklist for ML-based scientific claims; 19 researchers; EQUATOR-listed | ML scientific reporting — cross-disciplinary | Yes |
| G6 | Flanagin A, et al. (2023). Guidance for authors, peer reviewers, and editors on use of AI, language models, and chatbots. JAMA. | 2023 | Editorial Policy | **Tier 4** | JAMA Network editorial guidance banning AI authorship; requires disclosure in methods or acknowledgments | AI authorship and peer review — JAMA Network | — |
| G6 | NEJM Group. (2024). NEJM AI policy. | 2024 | Editorial Policy | **Tier 4** | NEJM dual disclosure policy prohibiting AI authorship and AI-generated images | AI authorship and disclosure — NEJM | — |
| G6 | Nature Portfolio. (2026). Nature AI Policy: What is allowed and required. | 2026 | Publisher Policy | **Tier 4** | Nature strict rules prohibiting AI authorship and mandating detailed disclosure | AI authorship and disclosure — Nature journals | — |
| G6 | Elsevier. (2024). Generative AI policies for journals. | 2024 | Publisher Policy | **Tier 4** | Publisher-wide rules prohibiting AI authorship; authors must verify AI outputs | AI authorship — Elsevier publisher policy | — |
| G6 | Wiley. (2024). AI guidelines for researchers. | 2024 | Publisher Policy | **Tier 4** | Comprehensive publisher guidance on research integrity and transparent AI disclosure | AI disclosure and integrity — Wiley | — |
| G6 | STM Association. (2025). Recommendations for a classification of AI use in academic manuscript preparation. | 2025 | Trade Body Guidance | **Tier 4** | Harmonises terminology across publisher policies on AI assistance | AI classification and terminology harmonisation | — |
| G6 | CMAJ. (2023). A new policy on the use of artificial intelligence tools for manuscripts submitted to CMAJ. | 2023 | Editorial Policy | **Tier 4** | Journal policy reinforcing ICMJE standards on AI authorship prohibition | AI authorship policy — CMAJ | — |
| G6 | RSNA Publications. (2025). Editorial policies on AI-assisted technologies. | 2025 | Editorial Policy | **Tier 4** | RSNA ban on AI authorship; requires adherence to STARD and CONSORT checklists | AI authorship and reporting — radiology journals | — |
| G6 | Lima CMD, Gross PdSC. (2026). Politicas de uso de Inteligencia Artificial por periodicos da area da Saude. Abec Meeting. | 2026 | Conference Review | **Contextual** | Descriptive review of AI usage policies in Brazilian health journals; conference venue; preliminary scope | AI policy in Brazilian health journals | — |
| G6 | Leung TI, et al. (2023). Best practices for using AI tools as an author, peer reviewer, or editor. JMIR. | 2023 | Guidance Document | **Tier 4** | Expert guidance on AI disclosure and peer review confidentiality; policy-type content | AI best practices for authors, reviewers, and editors | — |
| G6 | British Journal of Anaesthesia. (2026). Responsible use of LLMs in manuscript authorship, peer review, and editorial processes. PMID:41748337. | 2026 | Specialty Guidance | **Tier 4** | Specialty-specific guidance on responsible LLM use across authorship and peer review | LLM use in anaesthesia journal publishing | — |
| G6 | Cleland J, et al. (2025). When and how to disclose AI use in academic publishing: AMEE Guide No. 192. Medical Teacher. | 2025 | Professional Association Guideline | **Tier 4** | Practical framework for health researchers to ethically report AI in scholarly writing | AI disclosure in health education publishing | — |
| G6 | Fontenot J. (2025). Artificial intelligence disclosure in academic nursing: Framework for editorial policy. Nurse Author and Editor. | 2025 | Professional Publication Framework | **Tier 4** | Nursing-specific AI authorship prohibition framework based on COPE principles | AI disclosure in nursing scholarly communication | — |
| G6 | Schrager S, et al. (2025). Use of AI in family medicine publications: Joint editorial from journal editors. Journal of the American Board of Family Medicine. | 2025 | Joint Editorial Policy | **Tier 4** | Unified editorial statement on standardising AI disclosure in family medicine publications | AI disclosure in family medicine | — |
| G6 | UNESCO. (2021). Ethics of artificial intelligence. UNESCO. | 2021 | Global Framework | **Tier 4** | Global human rights and ethical guidelines for AI development and deployment | Global AI ethics — UNESCO | — |
| G6 | European Association of Science Editors. (2023). Recommendations on the use of AI in scholarly communication. EASE. | 2023 | Professional Association Guidance | **Tier 4** | Advises journals on formulating AI policies and ethical scholarly communication | AI in scholarly communication — EASE | — |
| G6 | Ganjavi C, Eppler MB, et al. (2024). Bibliometric analysis of publisher and journal instructions on generative AI. BMJ, 384, e077192. | 2024 | Bibliometric Analysis | Contextual | Finds 95.7% of top 100 journals prohibit AI authorship; contextual evidence supporting narrative synthesis | AI authorship policy prevalence — bibliometric assessment | — |
| G6 | Doskaliuk B, et al. (2025). AI in peer review: Enhancing efficiency while preserving integrity. Trends in Scholarly Publishing. | 2025 | Review | Contextual | Explores ethical challenges of AI in peer review; contextual evidence | AI in peer review integrity and oversight | — |
| G6 | da Veiga A. (2025). Ethical guidelines for generative AI and AI-assisted tools in scholarly publishing: Thematic analysis. Science Editing. | 2025 | Systematic Thematic Analysis | Contextual | Systematic thematic analysis of top 10 publishers policies; contextual evidence | AI publishing ethics — thematic synthesis | — |
| G6 | Yoo JH. (2025). Defining boundaries of AI use in scientific writing: Comparative review of editorial policies. Journal of Korean Medical Science. | 2025 | Review | Contextual | Compares editorial policies across specialties; contextual evidence | AI editorial policy comparison — cross-specialty | — |
| G6 | Gurnal P, Rana L. (2025). Artificial intelligence and publishing ethics: A narrative review and SWOT analysis. Cureus. | 2025 | Review | Contextual | SWOT analysis evaluating AI publishing ethics; contextual evidence | AI publishing ethics SWOT analysis | — |
| G6 | Lin Z. (2024). Towards an AI policy framework in scholarly publishing. Trends in Cognitive Sciences. | 2024 | Perspective Paper | Contextual | Proposes AI policy framework; single-author perspective; contextual evidence | AI policy framework for scholarly publishing | — |
| G6 | Bains V, Bhawal U. (2025). Biomedical research publication in the age of artificial intelligence. Journal of Healthcare Research and Education. | 2025 | Review | Contextual | Reviews AI innovation and editorial consensus; contextual evidence | AI and research integrity — biomedical publishing | — |
| G6 | Quagliato L, et al. (2024). Artificial intelligence in mental health care: Less hype, more accountability. | 2024 | Editorial Commentary | Contextual | Discusses risk-based oversight for AI in mental health; contextual evidence | AI accountability in mental health governance | — |
| G6 | Cheng K, Wu H. (2024). Policy framework for the utilisation of generative AI. Critical Care. | 2024 | Review | Contextual | Summarises publisher guidelines for generative AI; contextual evidence | Generative AI policy summary for researchers | — |
| G6 | Kargl M, et al. (2022). Literature review on ethics for AI in biomedical research and biobanking. | 2022 | Literature Review | Contextual | Reviews AI ethics in biomedical research; contextual evidence | AI ethics in biomedical research and biobanking | — |
| G6 | Bhargava M, et al. (2024). Artificial intelligence in biomedical research and publications. Indian Journal of Community Medicine. | 2024 | Review | Contextual | Reviews global policies emphasising human authorship; contextual evidence | AI authorship ethics — global policy review | — |
| G6 | Colcelli V, Burzagli L. (2023). Ethical and regulatory frameworks for AI in clinical research. | 2023 | Review | Contextual | Details European regulatory landscape including AI Act; contextual evidence | European AI regulatory framework for research | — |
| G6 | Yadav S. (2024). Enhancing research integrity and publication ethics: Analysis of the latest ICMJE recommendations. | 2024 | Review | Contextual | Analyses ICMJE recommendations for AI and research integrity; contextual evidence | ICMJE AI recommendations analysis | — |
| G6 | Kanwal R, Tayyub U, Awan WA. (2025). AI tools in rehabilitation research: A call for full disclosure. Rehabilitation Journal. | 2025 | Commentary | **Contextual** | Advocates mandatory disclosure and standardisation of AI reporting in rehabilitation | AI disclosure in rehabilitation research | — |
