# Supplementary material for: Transparency, accountability, and scholarly integrity: A systematic narrative review of artificial intelligence governance frameworks in healthcare publication
Source: PLOS Digit Health. 2026 Oct 20;5(10):e0001783. doi: 10.1371/journal.pdig.0001783 (PMC13682662; doi:10.1371/journal.pdig.0001783)
Supplement: Supplementary file 5 [file pdig.0001783.s005.docx]

**Supporting Information — S5 File - Systematic Search Script**

**Overview**

**Language:** Python 3.9+

**Dependencies:** requests, pandas, openpyxl (install via pip install requests pandas openpyxl)

**Usage:** python ai_guidelines_systematic_search_v2.py

**Optional:** Set NCBI_API_KEY environment variable for higher API rate limits (10 req/sec vs 3 req/sec)

**Output:** Files written to ./search_results/ — includes all_results_raw.xlsx, deduplicated.xlsx, screening_sheet.xlsx, prisma_counts.txt, search_strings.txt

**Source:** The relevance scoring algorithm (compute_relevance_score() function) is documented in S2 File.

**Script**

#!/usr/bin/env python3

"""

Systematic Search Tool — AI Reporting Guidelines in Healthcare Publications

============================================================================

Searches PubMed (via NCBI E-utilities) and EQUATOR Network for reporting

guidelines, frameworks, checklists, and editorial policies on AI use in

healthcare research and publication.

Threshold selected based on pilot testing. No formal external validation was performed. Script

for additional candidates. Covers 14 thematic search blocks.

Usage:

pip install requests pandas openpyxl

python ai_guidelines_systematic_search.py

Optional: set your NCBI API key for higher rate limits (10 req/sec vs 3):

export NCBI_API_KEY="your_key_here"

(Get a free key at: https://www.ncbi.nlm.nih.gov/account/)

Output files (in ./search_results/):

- search_log.txt	Full audit trail of all searches run

- all_results_raw.xlsx	All retrieved records before deduplication

- deduplicated.xlsx	Unique records after deduplication by PMID/DOI/title

- screening_sheet.xlsx	Ready-to-use title/abstract screening worksheet

- prisma_counts.txt	PRISMA-style record counts for your flow diagram

- search_strings.txt	All Boolean strings used (for Methods appendix)

"""

import os

import re

import time

import json

import hashlib

import logging

import requests

import unicodedata

from datetime import datetime

from pathlib import Path

import xml.etree.ElementTree as ET

try:

import pandas as pd

except ImportError:

raise SystemExit("Install pandas first: pip install pandas openpyxl")

\# ── Configuration ─────────────────────────────────────────────────────────────

OUTPUT_DIR = Path("./search_results")

OUTPUT_DIR.mkdir(exist_ok=True)

NCBI_API_KEY = os.environ.get("NCBI_API_KEY", "")

NCBI_BASE	= "https://eutils.ncbi.nlm.nih.gov/entrez/eutils"

EQUATOR_URL = "https://www.equator-network.org/reporting-guidelines/"

\# Date range — adjust as needed

DATE_FROM = "2018/01/01"

DATE_TO	= "2026/05/19"

\# Rate limiting (seconds between requests)

DELAY_WITH_KEY	= 0.12	# ~8 req/sec, safely under the 10/sec limit

DELAY_WITHOUT_KEY = 0.40	# ~2.5 req/sec, safely under the 3/sec limit

DELAY = DELAY_WITH_KEY if NCBI_API_KEY else DELAY_WITHOUT_KEY

logging.basicConfig(

level=logging.INFO,

format="%(asctime)s %(levelname)-8s %(message)s",

handlers=[

logging.FileHandler(OUTPUT_DIR / "search_log.txt", encoding="utf-8"),

logging.StreamHandler(),

],

)

log = logging.getLogger(__name__)

\# ── Search strings ─────────────────────────────────────────────────────────────

\# Each entry: (label, pubmed_query)

\# Designed to be broad at first, then targeted by concept cluster.

\# All strings use MeSH terms where available + free-text synonyms.

SEARCH_STRINGS = [

\# ── BLOCK 1: Core concept — AI + reporting/guidelines ─────────────────────

(

"Core_AI_reporting",

(

'("artificial intelligence"[MeSH] OR "machine learning"[MeSH] OR '

'"deep learning"[tw] OR "neural network"[tw] OR '

'"large language model*"[tw] OR "LLM"[tw] OR "generative AI"[tw] OR '

'"foundation model*"[tw]) '

'AND '

'("guideline*"[tw] OR "reporting standard*"[tw] OR "checklist*"[tw] OR '

'"reporting item*"[tw] OR "reporting framework*"[tw] OR '

'"consensus statement*"[tw] OR "Delphi"[tw] OR "EQUATOR"[tw]) '

'AND '

'("healthcare"[tw] OR "health care"[tw] OR "clinical"[tw] OR '

'"biomedical"[tw] OR "medicine"[tw] OR "nursing"[tw] OR '

'"public health"[tw])'

),

),

\# ── BLOCK 2: Publication ethics + AI ──────────────────────────────────────

(

"Publication_ethics_AI",

(

'("artificial intelligence"[MeSH] OR "machine learning"[MeSH] OR '

'"generative AI"[tw] OR "large language model*"[tw] OR "ChatGPT"[tw] OR '

'"GPT"[tw] OR "LLM"[tw]) '

'AND '

'("authorship"[tw] OR "publication ethics"[tw] OR "research integrity"[tw] OR '

'"peer review"[tw] OR "disclosure"[tw] OR "transparency"[tw] OR '

'"editorial polic*"[tw] OR "journal polic*"[tw]) '

'AND '

'("guideline*"[tw] OR "recommendation*"[tw] OR "framework*"[tw] OR '

'"standard*"[tw] OR "policy"[tw])'

),

),

\# ── BLOCK 3: Named EQUATOR-affiliated AI guidelines ────────────────────────

(

"Named_EQUATOR_guidelines",

(

'"CONSORT-AI"[tw] OR "SPIRIT-AI"[tw] OR "TRIPOD-AI"[tw] OR '

'"TRIPOD+AI"[tw] OR "PROBAST-AI"[tw] OR "PROBAST+AI"[tw] OR '

'"STARD-AI"[tw] OR "DECIDE-AI"[tw] OR "CLAIM"[tw] OR '

'"FUTURE-AI"[tw] OR "STANDING Together"[tw] OR "GAMER"[tw] OR '

'"CHART"[tw] OR "TRIPOD-LLM"[tw] OR "CANGARU"[tw] OR '

'"MI-CLAIM"[tw] OR "CHEERS-AI"[tw] OR "REFORMS"[tw] OR '

'"CLEAR"[tw] OR "METRICS"[tw] OR "CAIR"[tw] OR "GUIDE-AI"[tw] OR '

'"AIHGIe"[tw] OR "TREG-AI"[tw] OR "QUADAS-AI"[tw] OR '

'"R-AI-DIOLOGY"[tw] OR "PRIME"[tw] OR "STREAM-URO"[tw] OR '

'"MINIMAR"[tw] OR "SCARE"[tw] OR "PROCESS"[tw] OR "STROCSS"[tw]'

),

),

\# ── BLOCK 4: Systematic reviews of AI guidelines ──────────────────────────

(

"Systematic_reviews_of_AI_guidelines",

(

'("systematic review"[Publication Type] OR "scoping review"[tw] OR '

'"meta-analysis"[Publication Type]) '

'AND '

'("artificial intelligence"[MeSH] OR "machine learning"[MeSH]) '

'AND '

'("reporting guideline*"[tw] OR "reporting standard*"[tw] OR '

'"checklist*"[tw] OR "guideline quality"[tw] OR "AGREE"[tw])'

),

),

\# ── BLOCK 5: Predictive model reporting (TRIPOD family) ───────────────────

(

"Predictive_model_reporting",

(

'("prediction model*"[tw] OR "prognostic model*"[tw] OR '

'"diagnostic model*"[tw] OR "clinical prediction"[tw]) '

'AND '

'("machine learning"[MeSH] OR "artificial intelligence"[MeSH] OR '

'"deep learning"[tw]) '

'AND '

'("reporting"[tw] OR "transparency"[tw] OR "checklist"[tw] OR '

'"guideline"[tw])'

),

),

\# ── BLOCK 6: LLM-specific reporting ───────────────────────────────────────

(

"LLM_chatbot_reporting",

(

'("large language model*"[tw] OR "ChatGPT"[tw] OR "GPT-4"[tw] OR '

'"Bard"[tw] OR "Gemini"[tw] OR "Claude"[tw] OR "chatbot*"[tw]) '

'AND '

'("reporting"[tw] OR "guideline*"[tw] OR "checklist*"[tw] OR '

'"framework*"[tw] OR "standard*"[tw]) '

'AND '

'("health"[tw] OR "medical"[tw] OR "clinical"[tw])'

),

),

\# ── BLOCK 7: Radiology / imaging AI guidelines ────────────────────────────

(

"Radiology_imaging_AI",

(

'("radiology"[MeSH] OR "radiomics"[tw] OR "medical imaging"[tw] OR '

'"diagnostic imaging"[MeSH]) '

'AND '

'("artificial intelligence"[MeSH] OR "deep learning"[tw] OR '

'"machine learning"[MeSH]) '

'AND '

'("reporting guideline*"[tw] OR "checklist*"[tw] OR "CLAIM"[tw] OR '

'"CLEAR"[tw] OR "METRICS"[tw] OR "RQS"[tw] OR "STARD"[tw])'

),

),

\# ── BLOCK 8: Specialty-specific AI guidelines ─────────────────────────────

(

"Specialty_AI_guidelines",

(

'("artificial intelligence"[MeSH] OR "machine learning"[MeSH]) '

'AND '

'("dermatology"[tw] OR "cardiology"[tw] OR "urology"[tw] OR '

'"surgery"[tw] OR "ophthalmology"[tw] OR "pathology"[tw] OR '

'"oncology"[tw]) '

'AND '

'("reporting guideline*"[tw] OR "checklist*"[tw] OR '

'"reporting standard*"[tw])'

),

),

\# ── BLOCK 9: Fairness, bias, equity in AI reporting ───────────────────────

(

"Fairness_bias_AI_reporting",

(

'("algorithmic bias"[tw] OR "fairness"[tw] OR "equity"[tw] OR '

'"disparity"[tw] OR "representation"[tw]) '

'AND '

'("artificial intelligence"[MeSH] OR "machine learning"[MeSH]) '

'AND '

'("reporting"[tw] OR "guideline*"[tw] OR "framework*"[tw] OR '

'"standard*"[tw] OR "checklist*"[tw]) '

'AND '

'("health"[tw] OR "medical"[tw] OR "clinical"[tw])'

),

),

\# ── BLOCK 10: Health economic evaluation + AI ─────────────────────────────

(

"Health_economics_AI",

(

'("cost-effectiveness"[tw] OR "health economic*"[tw] OR '

'"economic evaluation"[tw] OR "CHEERS"[tw]) '

'AND '

'("artificial intelligence"[MeSH] OR "machine learning"[MeSH]) '

'AND '

'("reporting"[tw] OR "guideline*"[tw] OR "standard*"[tw])'

),

),

\# ── BLOCK 11: Editorial/publisher policies on AI ──────────────────────────

(

"Editorial_publisher_policies",

(

'("ICMJE"[tw] OR "WAME"[tw] OR "COPE"[tw] OR "editorial board"[tw] OR '

'"journal editor*"[tw] OR "publisher*"[tw]) '

'AND '

'("artificial intelligence"[MeSH] OR "generative AI"[tw] OR '

'"large language model*"[tw] OR "ChatGPT"[tw]) '

'AND '

'("policy"[tw] OR "guideline*"[tw] OR "recommendation*"[tw] OR '

'"disclosure"[tw] OR "authorship"[tw])'

),

),

\# ── BLOCK 12: Real-world evidence + AI reporting (catches ESMO GROW) ───────

(

"Real_world_evidence_AI",

(

'("real-world evidence"[tw] OR "real world data"[tw] OR '

'"observational stud*"[tw] OR "registry"[tw]) '

'AND '

'("artificial intelligence"[MeSH] OR "machine learning"[MeSH] OR '

'"deep learning"[tw]) '

'AND '

'("reporting"[tw] OR "guideline*"[tw] OR "standard*"[tw] OR '

'"framework*"[tw] OR "checklist*"[tw]) '

'AND '

'("oncology"[tw] OR "cancer"[tw] OR "clinical"[tw] OR '

'"healthcare"[tw])'

),

),

\# ── BLOCK 13: Multi-centre / clustered prediction model reporting ─────────

\# Catches TRIPOD-Cluster and similar extensions for complex study designs

(

"Clustered_prediction_models",

(

'("TRIPOD"[tw] OR "prediction model*"[tw] OR "prognostic model*"[tw]) '

'AND '

'("cluster*"[tw] OR "multi-centre"[tw] OR "multicenter"[tw] OR '

'"federated"[tw] OR "distributed"[tw] OR "external validation"[tw]) '

'AND '

'("reporting"[tw] OR "guideline*"[tw] OR "checklist*"[tw] OR '

'"transparency"[tw])'

),

),

\# ── BLOCK 14: AI governance / regulatory frameworks in health ──────────────

(

"AI_governance_regulatory",

(

'("governance"[tw] OR "regulation"[tw] OR "regulatory framework"[tw] OR '

'"AI Act"[tw] OR "FDA"[tw]) '

'AND '

'("artificial intelligence"[MeSH] OR "machine learning"[MeSH]) '

'AND '

'("health"[MeSH Subheading] OR "healthcare"[tw] OR "clinical"[tw]) '

'AND '

'("reporting"[tw] OR "guideline*"[tw] OR "transparency"[tw] OR '

'"accountability"[tw])'

),

),

]

\# ── PubMed helpers ────────────────────────────────────────────────────────────

def pubmed_search(query: str, date_from: str, date_to: str) -> list[str]:

"""Run ESearch and return list of PMIDs."""

params = {

"db": "pubmed",

"term": f"({query}) AND (\"{date_from}\"[PDAT]:\"{date_to}\"[PDAT])",

"retmax": 10000,

"retmode": "json",

"usehistory": "y",

}

if NCBI_API_KEY:

params["api_key"] = NCBI_API_KEY

try:

r = requests.get(f"{NCBI_BASE}/esearch.fcgi", params=params, timeout=30)

r.raise_for_status()

data = r.json()

pmids = data.get("esearchresult", {}).get("idlist", [])

count = data.get("esearchresult", {}).get("count", "?")

log.info(f" → ESearch returned {count} records ({len(pmids)} retrieved)")

return pmids

except Exception as e:

log.error(f" ESearch failed: {e}")

return []

def pubmed_fetch_details(pmids: list[str]) -> list[dict]:

"""Fetch article details for a list of PMIDs in batches."""

if not pmids:

return []

records = []

batch_size = 200

for i in range(0, len(pmids), batch_size):

batch = pmids[i : i + batch_size]

params = {

"db": "pubmed",

"id": ",".join(batch),

"retmode": "xml",

"rettype": "abstract",

}

if NCBI_API_KEY:

params["api_key"] = NCBI_API_KEY

try:

r = requests.get(f"{NCBI_BASE}/efetch.fcgi", params=params, timeout=60)

r.raise_for_status()

records.extend(parse_pubmed_xml(r.text))

log.info(f"	Fetched batch {i//batch_size + 1} ({len(batch)} records)")

except Exception as e:

log.error(f"	EFetch batch failed: {e}")

time.sleep(DELAY)

return records

def parse_pubmed_xml(xml_text: str) -> list[dict]:

"""Parse PubMed XML and extract key fields."""

try:

root = ET.fromstring(xml_text)

except ET.ParseError as e:

log.error(f"XML parse error: {e}")

return []

records = []

for article in root.findall(".//PubmedArticle"):

rec = {}

\# PMID

pmid_el = article.find(".//PMID")

rec["pmid"] = pmid_el.text if pmid_el is not None else ""

\# Title

title_el = article.find(".//ArticleTitle")

rec["title"] = _get_text(title_el)

\# Abstract

abstract_parts = article.findall(".//AbstractText")

rec["abstract"] = " ".join(_get_text(a) for a in abstract_parts).strip()

\# Authors

authors = []

for author in article.findall(".//Author"):

last = author.findtext("LastName", "")

fore = author.findtext("ForeName", "")

if last:

authors.append(f"{last} {fore}".strip())

rec["authors"] = "; ".join(authors[:6])

if len(authors) > 6:

rec["authors"] += " et al."

\# Journal

rec["journal"] = article.findtext(".//Journal/Title", "")

\# Year

year_el = article.find(".//PubDate/Year")

if year_el is None:

year_el = article.find(".//PubDate/MedlineDate")

rec["year"] = (year_el.text[:4] if year_el is not None else "")

\# Volume / Issue / Pages

rec["volume"] = article.findtext(".//Journal/JournalIssue/Volume", "")

rec["issue"] = article.findtext(".//Journal/JournalIssue/Issue", "")

rec["pages"] = article.findtext(".//MedlinePgn", "")

\# DOI

doi = ""

for id_el in article.findall(".//ArticleId"):

if id_el.get("IdType") == "doi":

doi = id_el.text or ""

break

rec["doi"] = doi

\# Publication types

pub_types = [pt.text for pt in article.findall(".//PublicationType") if pt.text]

rec["publication_types"] = "; ".join(pub_types)

\# MeSH terms

mesh = [d.findtext("DescriptorName", "") for d in article.findall(".//MeshHeading")]

rec["mesh_terms"] = "; ".join(filter(None, mesh))

\# PubMed URL

rec["pubmed_url"] = f"https://pubmed.ncbi.nlm.nih.gov/{rec['pmid']}/" if rec["pmid"] else ""

records.append(rec)

return records

def _get_text(el) -> str:

"""Recursively get all text from an XML element, preserving inner text."""

if el is None:

return ""

return "".join(el.itertext()).strip()

\# ── EQUATOR search ────────────────────────────────────────────────────────────

\# Known AI-related guidelines from EQUATOR (manual curation — EQUATOR has no API)

\# Each dict matches the same schema as PubMed records so they merge cleanly.

EQUATOR_KNOWN_AI_GUIDELINES = [

{

"pmid": "", "title": "CONSORT-AI Extension", "year": "2020",

"authors": "Liu X et al.", "journal": "BMJ; Nature Medicine",

"doi": "10.1136/bmj.m3164", "publication_types": "Reporting Guideline",

"mesh_terms": "Artificial Intelligence; Clinical Trials",

"abstract": "Extension of CONSORT for randomised trials of AI interventions.",

"equator": "Yes", "pubmed_url": "https://pubmed.ncbi.nlm.nih.gov/32938584/",

},

{

"pmid": "", "title": "SPIRIT-AI Extension", "year": "2020",

"authors": "Cruz Rivera S et al.", "journal": "BMJ; Nature Medicine",

"doi": "10.1136/bmj.m3210", "publication_types": "Reporting Guideline",

"mesh_terms": "Artificial Intelligence; Clinical Trial Protocols",

"abstract": "Extension of SPIRIT for protocols of clinical trials of AI.",

"equator": "Yes", "pubmed_url": "https://pubmed.ncbi.nlm.nih.gov/32934971/",

},

{

"pmid": "", "title": "TRIPOD+AI Statement", "year": "2024",

"authors": "Collins GS et al.", "journal": "BMJ",

"doi": "10.1136/bmj-2023-078378", "publication_types": "Reporting Guideline",

"mesh_terms": "Prediction Models; Machine Learning",

"abstract": "Updated TRIPOD for clinical prediction models using ML.",

"equator": "Yes", "pubmed_url": "https://pubmed.ncbi.nlm.nih.gov/38626948/",

},

{

"pmid": "", "title": "PROBAST+AI", "year": "2025",

"authors": "Moons KGM et al.", "journal": "BMJ",

"doi": "10.1136/bmj-2024-082505", "publication_types": "Appraisal Tool",

"mesh_terms": "Risk of Bias; Prediction Models; Machine Learning",

"abstract": "Updated risk of bias tool for prediction models using AI methods.",

"equator": "Yes", "pubmed_url": "",

},

{

"pmid": "", "title": "STARD-AI", "year": "2021",

"authors": "Sounderajah V et al.", "journal": "BMJ Open",

"doi": "10.1136/bmjopen-2021-047709", "publication_types": "Reporting Guideline",

"mesh_terms": "Diagnostic Accuracy; Artificial Intelligence",

"abstract": "Extension of STARD for diagnostic accuracy studies using AI.",

"equator": "Yes", "pubmed_url": "https://pubmed.ncbi.nlm.nih.gov/34385227/",

},

{

"pmid": "", "title": "DECIDE-AI", "year": "2022",

"authors": "Vasey B et al.", "journal": "Nature Medicine",

"doi": "10.1038/s41591-022-01815-1", "publication_types": "Reporting Guideline",

"mesh_terms": "Clinical Decision Support; Artificial Intelligence",

"abstract": "Reporting guideline for early clinical evaluation of AI decision support.",

"equator": "Yes", "pubmed_url": "https://pubmed.ncbi.nlm.nih.gov/35551246/",

},

{

"pmid": "", "title": "FUTURE-AI", "year": "2025",

"authors": "Lekadir K et al.", "journal": "BMJ",

"doi": "10.1136/bmj-2024-081554", "publication_types": "Consensus Guideline",

"mesh_terms": "Artificial Intelligence; Trustworthy AI",

"abstract": "International consensus guideline for trustworthy AI in healthcare.",

"equator": "Yes", "pubmed_url": "",

},

{

"pmid": "", "title": "GAMER Checklist", "year": "2025",

"authors": "Luo X et al.", "journal": "BMJ Evidence-Based Medicine",

"doi": "10.1136/bmjebm-2024-113218", "publication_types": "Reporting Guideline",

"mesh_terms": "Generative AI; Scientific Writing",

"abstract": "Reporting guideline for generative AI use in medical research.",

"equator": "Yes", "pubmed_url": "",

},

{

"pmid": "", "title": "CHART Checklist", "year": "2025",

"authors": "The CHART Collaborative", "journal": "JAMA Network Open",

"doi": "10.1001/jamanetworkopen.2025.30220", "publication_types": "Reporting Guideline",

"mesh_terms": "Chatbots; Large Language Models; Clinical Advice",

"abstract": "Reporting guideline for chatbot health advice studies.",

"equator": "Yes", "pubmed_url": "",

},

{

"pmid": "", "title": "TRIPOD-LLM", "year": "2025",

"authors": "Gallifant J et al.", "journal": "Nature Medicine",

"doi": "10.1038/s41591-024-03425-5", "publication_types": "Reporting Guideline",

"mesh_terms": "Large Language Models; Reporting",

"abstract": "Extension of TRIPOD for studies developing or evaluating LLMs.",

"equator": "Yes", "pubmed_url": "https://pubmed.ncbi.nlm.nih.gov/39779896/",

},

{

"pmid": "", "title": "CHEERS-AI", "year": "2024",

"authors": "Sagoo GS, Richardson M", "journal": "Int J Technol Assess Health Care",

"doi": "10.1017/S0266462324004756", "publication_types": "Reporting Guideline",

"mesh_terms": "Health Economics; Artificial Intelligence",

"abstract": "CHEERS extension for health economic evaluations of AI interventions.",

"equator": "Yes", "pubmed_url": "",

},

{

"pmid": "", "title": "REFORMS", "year": "2024",

"authors": "Kapoor S et al.", "journal": "Science Advances",

"doi": "10.1126/sciadv.adk3452", "publication_types": "Reporting Guideline",

"mesh_terms": "Machine Learning; Scientific Reproducibility",

"abstract": "32-item consensus checklist for ML-based scientific claims.",

"equator": "Yes", "pubmed_url": "",

},

{

"pmid": "", "title": "CLEAR — Radiomics", "year": "2023",

"authors": "Kocak B et al.", "journal": "Insights into Imaging",

"doi": "10.1186/s13244-023-01415-8", "publication_types": "Reporting Guideline",

"mesh_terms": "Radiomics; Reporting; Radiology",

"abstract": "CheckList for EvaluAtion of Radiomics Research; ESR/EuSoMII-endorsed.",

"equator": "Yes", "pubmed_url": "",

},

{

"pmid": "", "title": "METRICS — Radiomics", "year": "2024",

"authors": "Kocak B et al.", "journal": "Insights into Imaging",

"doi": "10.1186/s13244-024-01572-4", "publication_types": "Appraisal Tool",

"mesh_terms": "Radiomics; Quality Assessment",

"abstract": "30-item quality scoring tool for radiomics; EuSoMII-endorsed successor to RQS.",

"equator": "Yes", "pubmed_url": "",

},

{

"pmid": "", "title": "CAIR — Clinical AI Research Checklist", "year": "2021",

"authors": "Kunze KN et al.", "journal": "Acta Orthopaedica",

"doi": "10.1080/17453674.2021.1918389", "publication_types": "Reporting Guideline",

"mesh_terms": "Artificial Intelligence; Clinical Research",

"abstract": "Checklist to help clinicians read and evaluate AI research.",

"equator": "Yes", "pubmed_url": "",

},

{

"pmid": "", "title": "STANDING Together", "year": "2023",

"authors": "Alderman JE et al.", "journal": "Independent Consensus",

"doi": "", "publication_types": "Consensus Standard",

"mesh_terms": "Algorithmic Bias; Health Datasets; Transparency",

"abstract": "Consensus standards for health datasets to promote transparency and mitigate bias.",

"equator": "No", "pubmed_url": "",

},

{

"pmid": "", "title": "QUADAS-AI", "year": "2024",

"authors": "Deeks JJ et al.", "journal": "Annals of Internal Medicine",

"doi": "", "publication_types": "Appraisal Tool",

"mesh_terms": "Diagnostic Accuracy; Artificial Intelligence; Risk of Bias",

"abstract": "Extension of QUADAS-2 for systematic reviews of AI diagnostic accuracy studies.",

"equator": "Yes", "pubmed_url": "",

},

{

"pmid": "", "title": "GUIDE-AI (under development)", "year": "2024",

"authors": "EQUATOR Network", "journal": "Under development",

"doi": "", "publication_types": "Reporting Guideline (in development)",

"mesh_terms": "Clinical Practice Guidelines; Artificial Intelligence",

"abstract": "Reporting checklist on use of AI in the health guideline enterprise.",

"equator": "Yes", "pubmed_url": "",

},

{

"pmid": "", "title": "COPE Position Statement on AI in Publications", "year": "2023",

"authors": "COPE Council", "journal": "Committee on Publication Ethics",

"doi": "", "publication_types": "Policy Statement",

"mesh_terms": "Publication Ethics; Authorship; Artificial Intelligence",

"abstract": "COPE position on AI use in publications: AI cannot be listed as author; authors bear full responsibility for AI-generated content.",

"equator": "No", "pubmed_url": "https://publicationethics.org/cope-position-statements/ai-author",

},

{

"pmid": "", "title": "PRISMA-trAIce — Transparent Reporting of AI in Systematic Reviews", "year": "2025",

"authors": "Gottlieb M et al.", "journal": "Journal of Medical Internet Research",

"doi": "", "publication_types": "Reporting Guideline",

"mesh_terms": "Systematic Reviews; Artificial Intelligence; Transparent Reporting",

"abstract": "Checklist for transparent reporting of AI use in systematic literature reviews.",

"equator": "Yes", "pubmed_url": "",

},

{

"pmid": "", "title": "ESMO GROW — Guidance for Reporting Oncology Real-World Evidence", "year": "2023",

"authors": "ESMO", "journal": "Annals of Oncology",

"doi": "", "publication_types": "Reporting Guideline",

"mesh_terms": "Real-World Evidence; Oncology; Reporting",

"abstract": "ESMO guidance for reporting oncology real-world evidence including AI-assisted analyses.",

"equator": "No", "pubmed_url": "",

},

{

"pmid": "", "title": "TRIPOD-Cluster — Prediction Models in Clustered Data", "year": "2023",

"authors": "Wynants L et al.", "journal": "BMJ",

"doi": "", "publication_types": "Reporting Guideline",

"mesh_terms": "Prediction Models; Clustered Data; Multi-centre; Reporting",

"abstract": "Extension of TRIPOD for prediction models developed or validated in clustered (multi-centre) data.",

"equator": "Yes", "pubmed_url": "",

},

]

\# ── Deduplication ─────────────────────────────────────────────────────────────

def normalise_title(title: str) -> str:

"""Lowercase, strip punctuation, collapse whitespace for fuzzy title matching."""

t = unicodedata.normalize("NFKD", title).lower()

t = re.sub(r"[^a-z0-9 ]", " ", t)

return re.sub(r"\s+", " ", t).strip()

def deduplicate(records: list[dict]) -> list[dict]:

"""

Remove duplicates by:

1. Exact PMID match

2. Normalised DOI match

3. Near-identical title (first 80 normalised chars)

Returns deduplicated list, preserving first occurrence.

"""

seen_pmids: set[str] = set()

seen_dois: set[str] = set()

seen_titles: set[str] = set()

unique: list[dict] = []

for rec in records:

pmid = rec.get("pmid", "").strip()

doi	= rec.get("doi", "").strip().lower()

title = normalise_title(rec.get("title", ""))[:80]

if pmid and pmid in seen_pmids:

continue

if doi and doi in seen_dois:

continue

if title and title in seen_titles:

continue

if pmid:

seen_pmids.add(pmid)

if doi:

seen_dois.add(doi)

if title:

seen_titles.add(title)

unique.append(rec)

return unique

\# ── Relevance pre-screening ───────────────────────────────────────────────────

\# Keywords that strongly suggest a record is NOT a primary AI guideline paper

\# (e.g. clinical trials using AI as an intervention rather than guideline papers)

EXCLUDE_SIGNALS = [

"randomized controlled trial", "randomised controlled trial",

"phase ii", "phase iii", "drug trial", "vaccine", "surgery outcome",

"case report", "case series", "animal model", "murine", "mouse model",

"in vitro", "in vivo", "cell line",

]

INCLUDE_SIGNALS = [

"reporting guideline", "reporting checklist", "reporting standard",

"delphi", "consensus", "equator", "framework", "editorial policy",

"publication ethics", "transparency", "disclosure", "authorship",

"checklist", "appraisal tool", "quality assessment", "systematic review",

]

def compute_relevance_score(rec: dict) -> tuple[int, list[str]]:

"""

Returns (score: non-negative integer relevance score, matched_signals: list of matched terms).

score >= 3 → likely relevant; score < 3 → likely not relevant.

"""

haystack = (rec.get("title", "") + " " + rec.get("abstract", "")).lower()

score = 0

matched = []

for sig in INCLUDE_SIGNALS:

if sig in haystack:

score += 1

matched.append(f"+{sig}")

for sig in EXCLUDE_SIGNALS:

if sig in haystack:

score -= 2

matched.append(f"-{sig}")

\# Bonus: named AI guidelines in title

guideline_acronyms = [

"consort-ai", "spirit-ai", "tripod", "probast", "stard-ai",

"decide-ai", "claim", "future-ai", "gamer", "chart", "cair",

"reforms", "cheers-ai", "clear", "metrics", "minimar", "quadas",

"standing together", "guide-ai",

]

title_lower = rec.get("title", "").lower()

for acr in guideline_acronyms:

if acr in title_lower:

score += 2

matched.append(f"++acronym:{acr}")

break

return max(0, score), matched

\# ── Output builders ───────────────────────────────────────────────────────────

COLUMNS = [

"source", "search_block", "pmid", "title", "authors", "journal",

"year", "volume", "issue", "pages", "doi", "publication_types",

"mesh_terms", "equator", "abstract", "pubmed_url",

"relevance_score", "relevance_signals",

"screener_include", # Y / N / U (uncertain) — filled by human

"screener_notes",

"full_text_include", # Y / N — filled after full-text review

"full_text_notes",

"final_decision",

"thematic_group",

]

def build_row(rec: dict, source: str, block: str) -> dict:

score, signals = compute_relevance_score(rec)

row = {col: rec.get(col, "") for col in COLUMNS}

row["source"]	= source

row["search_block"]	= block

row["relevance_score"]	= score

row["relevance_signals"] = "; ".join(signals)

row["equator"]	= rec.get("equator", "")

row["screener_include"]	= ""

row["screener_notes"]	= ""

row["full_text_include"] = ""

row["full_text_notes"]	= ""

row["final_decision"]	= ""

row["thematic_group"]	= ""

return row

\# ── Main ──────────────────────────────────────────────────────────────────────

def main():

log.info("=" * 70)

log.info("Systematic search — AI reporting guidelines in healthcare")

log.info(f"Date range: {DATE_FROM} → {DATE_TO}")

log.info(f"NCBI API key: {'YES' if NCBI_API_KEY else 'NO (lower rate limit)'}")

log.info("=" * 70)

all_rows: list[dict] = []

prisma_counts: dict[str, int] = {}

\# ── PubMed searches ────────────────────────────────────────────────────────

for label, query in SEARCH_STRINGS:

log.info(f"\n[PubMed] Block: {label}")

pmids	= pubmed_search(query, DATE_FROM, DATE_TO)

records = pubmed_fetch_details(pmids)

prisma_counts[label] = len(records)

for rec in records:

all_rows.append(build_row(rec, source="PubMed", block=label))

time.sleep(DELAY)

\# ── EQUATOR curated list ───────────────────────────────────────────────────

log.info(f"\n[EQUATOR] Adding {len(EQUATOR_KNOWN_AI_GUIDELINES)} curated guidelines")

for rec in EQUATOR_KNOWN_AI_GUIDELINES:

all_rows.append(build_row(rec, source="EQUATOR_curated", block="EQUATOR"))

prisma_counts["EQUATOR_curated"] = len(EQUATOR_KNOWN_AI_GUIDELINES)

total_raw = len(all_rows)

log.info(f"\nTotal records before deduplication: {total_raw}")

\# ── Save raw ───────────────────────────────────────────────────────────────

df_raw = pd.DataFrame(all_rows, columns=COLUMNS)

raw_path = OUTPUT_DIR / "all_results_raw.xlsx"

df_raw.to_excel(raw_path, index=False, sheet_name="Raw results")

log.info(f"Raw results saved → {raw_path}")

\# ── Deduplicate ────────────────────────────────────────────────────────────

unique_rows = deduplicate(all_rows)

total_unique = len(unique_rows)

duplicates_removed = total_raw - total_unique

log.info(f"After deduplication: {total_unique} unique ({duplicates_removed} removed)")

df_dedup = pd.DataFrame(unique_rows, columns=COLUMNS)

dedup_path = OUTPUT_DIR / "deduplicated.xlsx"

with pd.ExcelWriter(dedup_path, engine="openpyxl") as writer:

df_dedup.to_excel(writer, index=False, sheet_name="All unique")

\# High-relevance subset

df_high = df_dedup[df_dedup["relevance_score"] >= 3].copy()

df_high.to_excel(writer, index=False, sheet_name="High relevance (score≥3)")

\# By year

for yr in sorted(df_dedup["year"].dropna().unique(), reverse=True):

if yr:

df_yr = df_dedup[df_dedup["year"] == yr]

sheet_name = f"Year {yr}"[:31]

df_yr.to_excel(writer, index=False, sheet_name=sheet_name)

log.info(f"Deduplicated results saved → {dedup_path}")

\# ── Screening sheet ────────────────────────────────────────────────────────

screening_cols = [

"pmid", "title", "authors", "journal", "year", "doi",

"publication_types", "equator", "relevance_score",

"abstract", "pubmed_url",

"screener_include", "screener_notes",

"full_text_include", "full_text_notes",

"final_decision", "thematic_group",

]

df_screen = df_dedup[screening_cols].sort_values(

["relevance_score", "year"], ascending=[False, False]

)

screen_path = OUTPUT_DIR / "screening_sheet.xlsx"

with pd.ExcelWriter(screen_path, engine="openpyxl") as writer:

df_screen.to_excel(writer, index=False, sheet_name="Title-Abstract screening")

\# Instructions sheet

instructions = pd.DataFrame({

"Instructions": [

"TITLE/ABSTRACT SCREENING GUIDE",

"",

"screener_include column:",

" Y = include (clearly meets criteria)",

" N = exclude (clearly does not meet criteria)",

" U = uncertain (proceed to full-text review)",

"",

"INCLUSION CRITERIA (all must be met):",

"1. Presents guidance, recommendations, reporting standards, editorial",

"	policies, ethical frameworks, or governance considerations related",

"	to AI/ML use in healthcare publication or scientific dissemination",

"2. Focused on healthcare, medicine, nursing, public health, or related",

"	clinical disciplines",

"3. Addresses AI applications in manuscript preparation, research reporting,",

"	peer review, authorship, transparency, reproducibility, or publication ethics",

"",

"EXCLUSION CRITERIA:",

"- Exclusively technical AI development without publication relevance",

"- AI applications unrelated to healthcare scholarship",

"- General AI ethics without implications for research dissemination",

"- Primary clinical studies (RCTs, cohort studies) that USE AI but do not",

" evaluate or propose reporting guidelines",

"- Animal studies, in vitro, non-healthcare contexts",

"",

"THEMATIC GROUPS (for final_decision column):",

"G1 - Predictive and diagnostic AI frameworks (CONSORT-AI, TRIPOD, etc.)",

"G2 - Sociotechnical and lifecycle governance (DECIDE-AI, FUTURE-AI, etc.)",

"G3 - Generative AI and LLM-specific (GAMER, CHART, TRIPOD-LLM, etc.)",

"G4 - Specialty-specific frameworks (radiology, cardiology, etc.)",

"G5 - Surgical and procedural guidelines",

"G6 - Editorial and publication policy (ICMJE, WAME, COPE, publishers)",

]

})

instructions.to_excel(writer, index=False, sheet_name="Instructions")

log.info(f"Screening sheet saved → {screen_path}")

\# ── PRISMA counts ──────────────────────────────────────────────────────────

prisma_text = [

"PRISMA-STYLE RECORD COUNTS",

"=" * 50,

f"Run date: {datetime.now().strftime('%Y-%m-%d %H:%M')}",

f"Date range searched: {DATE_FROM} to {DATE_TO}",

"",

"Records identified per search block:",

]

for block, count in prisma_counts.items():

prisma_text.append(f" {block:<40} {count:>6}")

prisma_text += [

"",

f"Total records identified (all sources): {total_raw}",

f"Duplicates removed: {duplicates_removed}",

f"Records after deduplication: {total_unique}",

f"Records with relevance score >= 3: {len(df_high)}",

"",

"Next steps:",

"1. Open screening_sheet.xlsx",

"2. Complete title/abstract screening (screener_include column: Y/N/U)",

"3. Retrieve full texts for all Y and U records",

"4. Complete full-text screening (full_text_include column: Y/N)",

"5. Record final_decision and thematic_group for all included records",

"6. Update PRISMA flow diagram with counts from each stage",

]

prisma_path = OUTPUT_DIR / "prisma_counts.txt"

prisma_path.write_text("\n".join(prisma_text), encoding="utf-8")

log.info(f"PRISMA counts saved → {prisma_path}")

\# ── Search strings for Methods appendix ───────────────────────────────────

strings_text = [

"SEARCH STRINGS — FOR METHODS / SUPPLEMENTARY APPENDIX",

"=" * 60,

f"Databases: PubMed (MEDLINE), EQUATOR Network (manual)",

f"Date range: {DATE_FROM} to {DATE_TO}",

"",

]

for i, (label, query) in enumerate(SEARCH_STRINGS, 1):

strings_text += [

f"String {i}: {label}",

"-" * 40,

query,

"",

]

strings_path = OUTPUT_DIR / "search_strings.txt"

strings_path.write_text("\n".join(strings_text), encoding="utf-8")

log.info(f"Search strings saved → {strings_path}")

\# ── Summary ────────────────────────────────────────────────────────────────

log.info("\n" + "=" * 70)

log.info("SEARCH COMPLETE")

log.info(f" Total retrieved (raw): {total_raw}")

log.info(f" After deduplication: {total_unique}")

log.info(f" High relevance (score≥3): {len(df_high)}")

log.info(f" Output directory: {OUTPUT_DIR.resolve()}")

log.info("=" * 70)

log.info("\nNext step: open screening_sheet.xlsx and begin title/abstract screening.")

if __name__ == "__main__":

main()
