# Supplementary material for: Transparency, accountability, and scholarly integrity: A systematic narrative review of artificial intelligence governance frameworks in healthcare publication
Source: PLOS Digit Health. 2026 Oct 20;5(10):e0001783. doi: 10.1371/journal.pdig.0001783 (PMC13682662; doi:10.1371/journal.pdig.0001783)
Supplement: Supplementary file 6 [file pdig.0001783.s007.docx]

**Response to Reviewers**

Manuscript ID: PDIG-D-26-00944

Journal: PLOS Digital Health

Resubmission date: July 20, 2026

Dear Editor,

We are grateful to the editors and all three reviewers for their thorough and constructive engagement with our manuscript. The reviewers' comments have substantially strengthened the work, particularly in relation to methodological transparency, framework enumeration, and discussion of the governance acceleration observed since 2023. Below we provide a point-by-point response to every comment raised, presented in a two-column format with the verbatim comment alongside our detailed response and the specific change made.

| **Comment (verbatim)** | **Author Response and Change Made** |
| --- | --- |
| **Journal Requirements** | |
| JR1 — Figure file format  Please provide separate figure files in .tif or .eps format. | All three figures have been converted to .tif format at 300 dpi and are submitted as separate files alongside this revision. Changes in figures were made to address the other reviewer’s comments, as well as figure 3 was deleted as it was repeated info across tables. |
| JR2 — Supporting Information legends  We have noticed that you have uploaded Supporting Information files, but you have not included a list of legends. | A full list of Supporting Information legends has been added to the manuscript after the References section, as required. Each file (S1–S5; S5 File contains the Python search script) is listed with its complete legend. |
| JR3 — Data availability: systematic review tables  Please ensure your manuscript or supplementary information includes: a numbered table of all studies identified in the literature search including excluded studies with exclusion reasons; a full data extraction table; an explanation of how missing data were handled. | We thank the editor for clarifying the journal’s data-availability expectations. We have revised the manuscript and supplementary materials to address these points as fully as permitted by the design and records of this systematic narrative review.  **Excluded records and reasons: Our search identified 35,847 records, of which 30,276 remained after deduplication. A pre-specified relevance-scoring process excluded 29,388 database records that did not meet the minimum threshold for progression to human screening. These records were not assessed individually at the document level. The remaining 888 records underwent human screening against the predefined eligibility criteria, and 788 were excluded, and the reasons for exclusions were: Focused exclusively on technical AI development without publication relevance; Addressed AI applications unrelated to healthcare scholarship; Discussed general AI ethics without implications for research dissemination. However, as this review was designed as a systematic narrative review of governance instruments, reporting guidance, and policy documents, exclusion decisions during human screening were recorded according to the predefined exclusion categories rather than through an individual paper-level exclusion log. Consequently, a retrospective table assigning a specific exclusion reason to each of the 788 records cannot be produced reliably without reconstructing decisions that were not recorded at that level. We believe that retrospectively assigning reasons would risk introducing inaccurate or unverifiable information.**  To maximize transparency, we have provided the complete PRISMA flow diagram in S3 File and have clarified in the Methods the exclusion categories applied during screening. Records were excluded when they: (i) addressed technical AI development without implications for research reporting, publication, or dissemination; (ii) discussed AI ethics or policy without providing actionable governance or reporting guidance; (iii) did not relate to healthcare, medicine, nursing, public health, or biomedical science; or (iv) represented duplicate or superseded versions of included instruments. We have also added the absence of an individual record-level exclusion log as a methodological limitation. We respectfully request that the flow diagram, predefined exclusion criteria, and explicit acknowledgement of this limitation be accepted in place of a retrospectively reconstructed exclusion table.  **Data extraction table: The complete extraction dataset used for this review is provided in S4 File. Because the review synthesized governance instruments, editorial policies, and reporting guidance rather than primary empirical studies, the extraction framework was intentionally focused on the characteristics required for the narrative and descriptive synthesis. For each of the 100 included publications, S4 reports the full citation, publication year, evidence tier, document type, primary governance function, target context, and EQUATOR registration status. These variables constitute the full structured dataset used to generate the findings reported in the manuscript. We have revised the Methods to clarify the scope of the extraction framework and its alignment with the review objectives.**  **Missing information:** The review did not extract participant-level data, effect estimates, clinical outcomes, or other numerical variables requiring statistical approaches to missing data. Where a source did not report information required for one of the predefined extraction fields, the information was retained as “not reported”; fields that were not relevant to a particular document type were treated as “not applicable.” No values were imputed, and no unreported information was inferred. This procedure has now been stated explicitly in the revised Methods. |
| JR4 — Data availability statement typo and specificity  Data availability statement: "All data in available within the manuscript and its materials" — please confirm all raw data are available and specify where. | We apologise for the typographical error. The statement has been corrected and expanded to explicitly reference all supplementary files. The revised statement reads: "All data underlying the findings of this review are available within the manuscript and its Supporting Information files. The complete list of 100 included publications with extracted characteristics is provided in S4 File. Search strings are provided in S1 File. The relevance-scoring algorithm is documented in S2 File. PRISMA-style record counts at each screening stage are provided in S3 File (a record-count table; individual exclusion reasons were not logged at paper level, as explained above)." |
| **Reviewer 1 — Major Concerns** | |
| R1-M1 — Framework count discrepancy  The abstract and Results state that 46 named frameworks were identified across six groups, and Table 1/Fig 3 report group sizes of G1=9, G2=5, G3=9, G4=8, G5=3, G6=7. These sum to 41, not 46. This discrepancy needs to be resolved and the count corrected throughout. | We thank the reviewer for this careful check, which identified a genuine inconsistency in the submitted manuscript. Cross-referencing all named frameworks listed in Table 1 and Figure 3 confirms that the reviewer’s count of 41 is correct: G1=9: CONSORT-AI, SPIRIT-AI, TRIPOD+AI, PROBAST+AI, STARD-AI, CLAIM/CLAIM-2024 (two publications, one framework), QUADAS-AI, CHEERS-AI, MI-CLAIM; G2=5 (DECIDE-AI, FUTURE-AI, STANDING Together, AIHGIe 2.0, TREG-AI); G3=9 (GAMER, CHART, TRIPOD-LLM, CANGARU [Cacciamani GE et al. 2023, arXiv:2307.08974, Tier 3], REFINE, MEDAI-LLM-SUMM, MIRACLE, PRISMA-trAIce, COREQ-LLM); G4=8 (PRIME/PRIME 2.0, STREAM-URO, R-AI-DIOLOGY, CLEAR Derm, CLEAR Radiomics, METRICS Radiomics, CAIR, LLM Radiology Delphi); G5=3 (SCARE, PROCESS, STROCSS); G6=7 (ICMJE/WAME policies, COPE, NIH policies [2023/2024], WHO guidance, MINIMAR, KJA Comprehensive Checklist, REFORMS [Tier 1]). The figure of “46” stated in the abstract, Results, and Table 1 heading was erroneous, it appears to have been a forward-looking count that included instruments described in the Phase 4 narrative text but not yet consolidated into Table 1 during final revision. We have corrected all instances of “46” to “41” throughout the abstract, Results section, Table 1 heading, and Figure 3 caption. |
| R1-M2 — Relevance-scoring algorithm transparency and validation  The manuscript does not provide sufficient evidence that the algorithm was validated or that potentially relevant frameworks were not systematically excluded. Information regarding algorithm development, validation procedures, sensitivity testing, and the likelihood of missed records is essential. | We appreciate this important methodological concern and have substantially expanded the Methods section to address it. The algorithm was developed iteratively by the research team as follows: (1) Development — 16 inclusion signal terms were pre-specified based on the review objectives and mapped to title/abstract text fields; each matched term contributing +1 point; records scoring ≥3 were prioritised for human screening. This threshold was selected because it required simultaneous co-occurrence of at least three distinct domain signals, substantially reducing the probability of topically irrelevant records reaching the screening stage. (2) Scope and acknowledged limitation — the algorithm prioritised precision over sensitivity by design: it was intended to create a manageable human-screening set from 30,276 records, not to serve as the primary eligibility filter. All eligibility decisions were made by human screeners on the 888 prioritised records; the algorithm performed pre-screening only. We acknowledge that records scoring below the threshold may include relevant instruments, and this is explicitly stated as a limitation in the revised manuscript. (3) Formal external validation of the algorithm was not performed, which we acknowledge as a limitation. To partially address this concern, we note that the manual EQUATOR Network search provided an independent pathway for framework identification not dependent on the algorithm, and several key instruments (e.g., DECIDE-AI, STANDING Together) were captured through EQUATOR independently of PubMed scoring. The full algorithm documentation, including all 16 inclusion signal terms and 15 exclusion signal terms and scoring variables, is provided in Supporting Information S2 File. The revised Methods section now makes these points explicit. |
| R1-M3 — Derivation of six groups and four phases not described.  The four governance phases and six thematic groups are presented as findings, but no method is described for how these categories were derived (e.g., inductive coding, consensus discussion, predefined taxonomy) or how disagreements were resolved. | This is a valid methodological comment and we have added explicit description of the derivation process to the Data Synthesis subsection. The six thematic groups and four governance phases were derived inductively within this review: as each of the 100 included publications was extracted and characterised, it was mapped to an emergent framework category based on its primary governance function, target study design, and institutional origin. Initial groupings were proposed by the lead author, reviewed iteratively by co-authors during the synthesis phase, and finalised by team consensus. Where a source addressed frameworks spanning multiple groups, it was assigned to the group corresponding to its primary governance focus. The four governance phases were identified through chronological content analysis during narrative synthesis: all 100 publications were ordered by publication year and reviewed for shifts in governance priorities, instrument type, and institutional origin. Phase boundaries were identified by the lead author, reviewed by all co-authors, and finalised by consensus discussion. No formal disagreement resolution procedure (e.g., third-party arbitration) was required, as co-author consensus was reached in all cases. This process is now explicitly described in the Data Synthesis section. |
| R1-M4 — Tier assignment process not described  The process by which frameworks were assigned to tiers requires greater transparency. The criteria for tier assignment are described conceptually, but the manuscript does not indicate whether classifications were performed independently by multiple reviewers, whether disagreements were resolved through consensus, or whether any assessment of inter-rater reliability was conducted. | We thank the reviewer for this important point. The tier assignment process has been explicitly described in the Evidence Stratification subsection. Tier criteria were pre-specified before data extraction (Tier 1 = formal EQUATOR-listed guidelines developed through Delphi and registered on EQUATOR; Tier 2 = peer-reviewed consensus frameworks in indexed journals without EQUATOR registration; Tier 3 = emerging, preliminary, or single-journal instruments; Tier 4 = editorial, institutional, and regulatory policies). Tier assignment was performed by the lead author (A.R.S.) and verified by a second author (V.S.S.) for the 69 governance instruments and policies (of the 100 included publications). Disagreements were resolved through consensus discussion among the full author team. Formal inter-rater reliability statistics (e.g., Cohen's kappa) were not calculated, which we acknowledge as a limitation; however, we note that the pre-specified, criterion-referenced nature of the tier definitions — particularly the objective Tier 1 criterion of EQUATOR registration — substantially reduced the degree of interpretive judgement required for the majority of assignments. The small number of borderline cases (predominantly instruments straddling Tier 2 and Tier 3) were resolved through team consensus and are noted in S4 File. The final tier distribution across 69 governance instruments is: Tier 1=20, Tier 2=9, Tier 3=15, Tier 4=25. |
| **Reviewer 1 — Minor Concerns** | |
| R1-m1 — Date inconsistency  The abstract/title page describe the search as covering "1 January 2018 to 19 May 2026," while Section 2.2 states searches were run "in May 2026, covering January 2018 to May 2026." These dates should be reconciled. | The reviewer is correct. The final search was executed in 19 May 2026 (not April 2026), covering 1 January 2018 to 19 May 2026, as stated in the abstract and introduction. This has been corrected to "May 2026" throughout the manuscript, consistent with the search execution date confirmed by the research team and documented in S1 File. Additionally, STARD-AI has been reclassified from Tier 1 to Tier 3, as EQUATOR lists the STARD-AI extension as under development at the search date. This reduces the Tier 1 count from 21 to 20 and increases the Tier 3 count from 14 to 15 across the 69 governance instruments. |
| R1-m2 — Author name discrepancy  The author name appears as "Monina Lino" on the title/metadata page but "Monica Lino" on the manuscript itself. Please confirm the correct spelling. | "Monica Lino" is the correct spelling of the author's name. The appearance of "Monina Lino" on the title/metadata page is a typographical error. This has been corrected in the submission system and in the manuscript title page. |
| R1-m3 — Data availability statement  The statement "All data in available within the manuscript and its materials" contains a typo and does not specify where within the manuscript or supplementary materials the underlying dataset can be found. | The typographical error ("in available" → "is available") has been corrected and the statement expanded to explicitly reference each Supporting Information file, as described in our response to JR4 above. |
| **Reviewer 2 — Comments** | |
| R2-1 — Justification for separating G5 (surgical) from G4 (specialty)  Why are "surgical reporting updates" (G5) separated from "specialty-specific extensions" (G4)? No reasons are given. This should be explained. | We thank the reviewer for this question. The separation of G5 (Surgical and Procedural Reporting Updates) from G4 (Specialty-Specific AI Frameworks) reflects a meaningful governance distinction rather than an arbitrary categorisation. Group 4 frameworks are AI-specific instruments developed for particular diagnostic domains (radiology, dermatology, urology, cardiovascular imaging); they address AI model performance, imaging workflows, and specialty-specific technical requirements. Group 5 frameworks, by contrast, are updates to established surgical case reporting standards (SCARE, PROCESS, STROCSS) that integrate AI disclosure requirements into pre-existing procedural documentation structures. These represent a qualitatively different governance activity, adapting existing non-AI reporting instruments rather than developing new AI-specific ones, and target procedural and intraoperative AI contexts distinct from the diagnostic and predictive focus of G4. This distinction is now explicitly articulated in the text, and a clarifying sentence has been added to the Framework Group Characteristics section. |
| R2-2 — No imaging versus non-imaging distinction within G4  It seems puzzling that there is no distinct separation between imaging frameworks (radiology/pathology) and other non-imaging frameworks. These groups should be more clearly defined and elucidated. | We appreciate this observation. We have added explicit notation within the G4 discussion to distinguish imaging-focused frameworks (CLAIM, CLEAR Radiomics, METRICS Radiomics, R-AI-DIOLOGY, RQS) from non-imaging specialty frameworks (STREAM-URO, PRIME, CLEAR Derm, CAIR). The text now notes that imaging frameworks constitute the largest subgroup within G4, reflecting the historical dominance of radiology in clinical AI development. We have also added a sentence in the Methods section (Data Synthesis) clarifying that within-group substructure was characterised narratively rather than as a separate grouping level, to maintain a parsimonious six-group taxonomy. We considered whether imaging warranted a dedicated seventh group, but judged that this would oversubdivide specialty instruments at the cost of structural clarity; this decision is now made explicit. |
| R2-3 — Six groups not listed in main body text  The complete list is not given in the main body of the text — although it is in the abstract. | The reviewer is correct. The six thematic groups were described individually in the Findings section but we did not list those collectively in the main text. A summary sentence listing all six groups with their full labels has been added to the opening paragraph of the Framework Group Characteristics section, consistent with their presentation in Table 1 and Figure 3. |
| R2-4 — Table 1 should use textual labels not group numbers  Table 1 would be much clearer if textual labels for the groups were provided instead of group numbers. | We agree and Table 1 has been revised so that each group header row displays the full group label alongside the group number (e.g., "G1 — Predictive and Diagnostic AI Frameworks") rather than the number alone. |
| R2-5 — Discussion of rapid 2023–2026 evolution and authority gradient  Some discussion of the timelines and the rapid evolution of the research would be a welcome addition to the review. The timing of the final phases plays directly into the authority gradient. | We have addressed this comment by adding a new paragraph to the Discussion section. The paragraph explicitly connects the accelerated timeline of governance activity since 2023 to the third governance tension (epistemic equivalence and the authority gradient): the unprecedented speed of LLM proliferation created institutional pressure to respond before formal EQUATOR-registered guideline development processes, which typically require 18–36 months of Delphi consensus, could be completed. The result is that the 2023–2026 phase is disproportionately populated by Tier 3 and Tier 4 instruments, which may give the appearance of a robust governance ecosystem while reflecting institutional urgency rather than methodological maturity. The paragraph contextualises this as a structural challenge for the field, rapid publication volume should not be conflated with governance consolidation. |
| **Reviewer 3 — Comments** | |
| R3-1 — ISO/IEC 42001 and NIST AI Risk Management Framework  A complete review of AI governance frameworks must account for both international standards and national/regional policy regimes. ISO/IEC 42001 and the NIST AI Risk Management Framework are foundational reference points. If the authors will exclude these frameworks, it should explicitly state that such frameworks exist but were not found in the search. | ISO/IEC 42001 (AI Management System Standard) and the NIST AI Risk Management Framework are indeed foundational governance instruments, and the reviewer is correct that their absence requires explicit explanation. These frameworks were not identified in our search for two reasons. First, neither instrument is indexed in PubMed (MEDLINE), which was our primary database; they are technical and regulatory standards, not peer-reviewed publications, and therefore fall outside PubMed's indexing scope. Second, while our Search Block 14 targeted AI governance and regulation broadly, it retrieved literature discussing these frameworks rather than the primary standards themselves, which are published by ISO and NIST respectively rather than in biomedical journals. Third, and most importantly, our eligibility criteria required that included sources provide actionable reporting items, structured recommendations, or defined governance principles specifically for healthcare research publication or scientific dissemination. ISO/IEC 42001 is an organisational management system standard addressing AI risk at the enterprise level; NIST AI RMF is a voluntary framework for AI risk taxonomy applicable across sectors. Neither provides reporting guidance for the publication of AI research findings, which is the scope of this review. We have added an explicit statement to the Limitations section acknowledging that organisational and technical AI standards (including ISO/IEC 42001 and NIST AI RMF) were outside the search scope and eligibility criteria, and that their relationship to publication-level governance warrants attention in future integrative reviews. |

We hope these revisions fully address the reviewers' concerns and that the manuscript is now suitable for publication in PLOS Digital Health. We remain available to provide any further clarification the editors or reviewers may require.
