# Supplementary material for: Transparency, accountability, and scholarly integrity: A systematic narrative review of artificial intelligence governance frameworks in healthcare publication
Source: PLOS Digit Health. 2026 Oct 20;5(10):e0001783. doi: 10.1371/journal.pdig.0001783 (PMC13682662; doi:10.1371/journal.pdig.0001783)
Supplement: Supplementary file 7 [file pdig.0001783.s008.docx]

**Response to Reviewers**

**Manuscript ID:** PDIG-D-26-00944R1

**Title:** Transparency, accountability, and scholarly integrity: a systematic narrative review of artificial intelligence governance frameworks in healthcare publication

**Journal:** PLOS Digital Health

**Corresponding author:** Amina Silva, Brock University, School of Nursing (asilva@brocku.ca)

Dear Dr. Zare, Dr. Razzaghi, and Dr. Celi,

Thank you for the opportunity to submit a further revision of this manuscript, and thank you to the reviewers for a careful second reading. We have incorporated all revisions into the clean manuscript and the tracked-changes file; our point-by-point responses below identify the corresponding manuscript sections. We note at the outset that one request — formal validation of the relevance-scoring algorithm (Comment 1.2) — could not be completed for this revision because the validation analyses were not performed; rather than report analyses that were not carried out, we have addressed this transparently as an explicit methodological limitation, as detailed below.

**Additional Editor Comments**

*Thank you for submitting the revised manuscript. A few reviewer comments still require clarification or correction.*

**Response:** We have addressed every remaining point from Reviewers #1 and #2 individually below. The substantive changes are: an explicit and strengthened treatment of the un-performed algorithm validation as a methodological limitation (Section 2.2 and Limitations); a new Methods subsection describing the derivation and rating of the governance domains shown in Figure 2 (Section 2.6); the reframing of the evidence tiers as categories of developmental maturity and governance authority rather than as a quality signal (Sections 2.5 and throughout); an explicit disclosure of AI tool use (Section 2.7); an explanation of the reduction from 102 to 100 included publications and reconciliation of the analytical denominators (Section 3.1); and consistent use of the term "epistemic conflation." All changes are marked in the tracked-changes file.

**Reviewer #1**

**Comment 1.1 (general assessment)**

*The revised manuscript addresses a timely and important topic and has improved substantially in methodological transparency, internal consistency and presentation. The authors have responded constructively to the reviewers, corrected numerical and editorial inconsistencies, clarified the derivation of the six thematic groups and four governance phases, expanded the description of the relevance-scoring algorithm, and strengthened the data-availability statement.*

**Response:** We thank the reviewer for this assessment and for the detailed guidance across both rounds of review. No change required.

**Comment 1.2 (validation of the relevance-scoring algorithm)**

*However, several important methodological issues remain. The authors should validate the algorithm using an independent set of relevant records and a representative sample of below-threshold records.*

**Response:** We agree that formal validation would strengthen the algorithm, and we want to be transparent: these validation analyses were not performed for this revision. Rather than report analyses that were not conducted, we have addressed this directly and prominently as a methodological limitation. Section 2.2 now states that a formal validation against an independent reference set of relevant records and a representative sample of below-threshold records was not undertaken, that the algorithm’s sensitivity and its false-negative rate below the threshold have therefore not been empirically quantified, and that the potential for relevant records scoring below the threshold to have been missed is acknowledged. The Limitations section has been strengthened accordingly, stating that this limits the demonstrable completeness of the search and that the screening results should be interpreted with this uncertainty in mind. We considered this the only appropriate course given that the analyses were not carried out; we would be glad to conduct and report a formal validation should the editors consider it essential for acceptance.

**Comment 1.3 (handling of manually identified EQUATOR records)**

*The manuscript should resolve the contradictory descriptions of whether manually identified EQUATOR records were independently retained or subjected to the same scoring threshold.*

**Response:** Corrected. The two descriptions have been made consistent. Both the first paragraph of Section 2.2 and the algorithm-detail paragraph now state that records identified through the EQUATOR manual search were entered into the same deduplication and relevance-scoring workflow as the PubMed records and were subject to the same threshold, with no exemption on the basis of source.

**Comment 1.4 (groups and phases as interpretive synthesis)**

*The derivation of the six thematic groups and four governance phases is now better explained, but these should be presented explicitly as an interpretive synthesis.*

**Response:** Agreed and adopted. Section 3.2 now states explicitly that the six thematic groups and four governance phases are an interpretive synthesis produced by the review team, not a pre-existing, externally derived, or validated taxonomy, and that alternative defensible groupings and boundary years are possible. The framing is carried into the Findings preamble, which now describes the results as organized by the review team into four interpretive themes.

**Comment 1.5 (methodology for the governance domains in Figure 2)**

*More importantly, the governance domains displayed in Figure 2 are not described in the methodology. The manuscript does not explain whether these domains were predefined or derived inductively, how they were operationally defined, or how the ratings of "primary," "moderate" and "minor" coverage were assigned. The unit of analysis, reviewers involved and process used to generate group-level ratings should be reported. Without this information, Figure 2 and the conclusions derived from it are not reproducible.*

**Response:** Agreed. A new Methods subsection, 2.6 "Governance domain coverage mapping (Figure 2)," has been added. It reports that the eight domains were predefined by the review team before extraction and fixed as extraction fields; it lists the eight domain names; it gives the operational definition of coverage (a domain recorded as present when an instrument contained at least one explicit reporting item, checklist element, or stated requirement addressing it, with general aspirational language recorded separately as partial coverage); it states the unit of analysis (the individual named framework, n=41, for extraction; the thematic group, n=6, for display); it describes the raters (coding by the lead author with independent verification by a second author and consensus resolution) and notes that inter-rater reliability was not calculated; and it gives the pre-specified decision rules converting instrument-level codes into group-level "primary," "moderate," and "minor" ratings. The subsection also states that the instrument-level domain codes underlying Figure 2 are provided in S4 File so the group-level ratings can be reproduced.

**Comment 1.6 (citation of Figure 1)**

*Figure 1 also does not appear to be explicitly referenced in the main text and should be cited when presenting the search and selection results.*

**Response:** Corrected. Section 3.1 now cites Figure 1 explicitly when presenting the search and selection results, describing it as the PRISMA flow diagram of the search and selection process.

**Comment 1.7 (evidence tiers as a quality signal)**

*The evidence tiers should not be described as the "primary quality signal" without a formal quality-appraisal process. They classify instruments according to development pathway, publication status and institutional provenance, but do not directly measure methodological quality, validity, representativeness or implementability. They would be more accurately presented as developmental-maturity, governance-authority or instrument categories.*

**Response:** Agreed; this was an overstatement and has been corrected. The phrase "primary quality signal" has been removed. Section 2.4 (data synthesis) now states that evidence tier assignment describes developmental maturity and governance authority rather than serving as a quality signal, and is not a substitute for formal quality appraisal. The opening of the tier-classification section (2.5) has been reframed to state that the tiers describe developmental maturity and governance authority derived from development pathway, publication status, and institutional provenance, and do not measure methodological quality, validity, representativeness, or implementability. The Tier 1 description now reads "strongest governance authority within the reporting-guideline ecosystem" rather than "strongest methodological authority."

**Comment 1.8 (reduction from 102 to 100 included sources)**

*The authors should also explain the reduction from 102 included sources in the original submission to 100 in the revision*

**Response:** Explained. A sentence has been added to Section 3.1 recording that, during revision, the included set was re-audited against the exclusion criteria and two records were removed as non-distinct versions of instruments already included, reducing the total from 102 to 100, and that all counts, denominators, tier totals, and supporting files were updated accordingly.

**Comment 1.9 (reconciliation of analytical denominators)**

*reconcile the different analytical denominators*

**Response:** Addressed. Section 3.1 now states the three denominators explicitly and flags that they should not be conflated: the 100 included publications for all source-level statements; the 69 governance instruments and policies for all tier-stratified analyses; and the 41 named frameworks for Table 1, the thematic group counts, and Figure 2.

**Comment 1.10 (inconsistent lead-author initials)**

*correct the inconsistent lead-author initials*

**Response:** Corrected. The lead author is now consistently "A.S." throughout; the occurrence of "A.R.S." in the tier-assignment paragraph has been changed.

**Comment 1.11 (disclosure of AI tool use)**

*provide an explicit disclosure concerning the use of AI tools in conducting or preparing the review*

**Response:** Added. A new subsection, 2.7 "Use of AI tools in the conduct and preparation of this review," states which review tasks were performed by the named authors, that a large language model was used only for language editing and for checking the internal consistency of numerical counts, that all AI-assisted output was verified against source records by the authors, that the authors take full responsibility for the content, and that no AI tool is listed as an author, consistent with ICMJE and COPE positions.

**Comment 1.12 (claims of comprehensiveness and global applicability)**

*and moderate claims of comprehensiveness and global applicability given the restricted search sources and limited representation of non-English and LMIC literature.*

**Response:** Agreed and moderated. In the Author Summary, the description of the included set is now bounded by the actual search ("identified within the boundaries of a PubMed and EQUATOR search"). In the Discussion, "mapping the governance ecosystem more comprehensively than previous reviews" is now "mapping a broader segment of the governance ecosystem than previous reviews, within the boundaries of a PubMed and EQUATOR search." In the Limitations, a closing sentence states that the review should be read as a structured map of the indexed, predominantly English-language governance literature rather than as a comprehensive or globally representative account, and that it should not be read as characterizing governance activity in low- and middle-income countries or non-English publishing systems.

**Reviewer #2**

*Thanks for this revised submission. My major concerns have been addressed, leaving only a few minor points that would benefit from clarification.*

**Response:** We thank Dr. Hochheiser for the constructive review across both rounds. The three remaining points are addressed below.

**Comment 2.1 (epistemic equivalence versus epistemic conflation)**

*The abstract and the text mention "the risk of epistemic equivalence". Later in Section 3.4.3, the term "epistemic conflation" is used. "Epistemic equivalence" is a bit of an unsatisfactory term, as it implies that the risk lies in different items being epistemically equivalent, which is not what the paper rather implies. Rather, I think the authors are saying that there is a risk of "inappropriate perceptions of epistemic equivalence" - that readers will consider items to have the same degree of support even when they do not. I suggest either using the term "epistemic conflation" throughout or rephrasing to refer to the perception of epistemic equivalence.*

**Response:** The reviewer is right, and we have taken the first suggested option. "Epistemic conflation" is now used throughout. The Abstract now refers to "the risk of epistemic conflation, whereby heterogeneous instruments are perceived as carrying equivalent authority when treated as interchangeable," and the Author Summary, the cross-cutting-tensions discussion, the Discussion, and the Conclusion have all been changed to match. Where the third governance tension is named, it now reads "epistemic conflation and the authority gradient."

**Comment 2.2 (ordering within Section 3.2)**

*Section 3.2 places the listing of the groups after the summary of those groups and their contents. This section might be clearer if the groups were moved to come before the summary.*

**Response:** Agreed. Section 3.2 now opens with the list of the six thematic groups (together with the statement that they are an interpretive synthesis), so that the groups are presented before the group-level summary and the discussion of their contents.

**Comment 2.3 (redundant Group column in Table 1)**

*This might be resolved during production, but in case it is not: the inclusion of a separate column for group in Table 1 takes up space without adding information, as the groups are given in separate table rows.*

**Response:** Noted and agreed. The group is already indicated by the grouped table rows, so the separate Group column is redundant. We are happy for it to be removed during production; we have retained it in the submitted file only so that the tracked-changes version remains legible, and we defer to the production team on the final layout.

**Reviewer #3**

*Thank you for covering this topic. It will serve as a good baseline for monitoring the progress of AI governance literature.*

**Response:** We thank the reviewer for this positive assessment. No change required.

We are grateful to the editors and reviewers for their time and for feedback that has materially improved the manuscript. We look forward to your decision.

Sincerely,

Amina Silva, on behalf of all authors
