# Supplementary material for: Transparency, accountability, and scholarly integrity: A systematic narrative review of artificial intelligence governance frameworks in healthcare publication
Source: PLOS Digit Health. 2026 Oct 20;5(10):e0001783. doi: 10.1371/journal.pdig.0001783 (PMC13682662; doi:10.1371/journal.pdig.0001783)
Supplement: Peer review history

# Peer review: Decision Letter 0

**DOI:** 10.1371/journal.pdig.0001783.r001

**Authors:** Hanieh Razzaghi, Amirhossein Zare

10 Jul 2026

Response to Reviewers Revised Manuscript with Track Changes Manuscript **Journal Requirements:**

1. Please provide separate figure files in .tif or .eps format.

For more information about figure files please see our guidelines:  LINK

https://journals.plos.org/digitalhealth/s/figures

https://journals.plos.org/digitalhealth/s/figures#loc-file-requirements

2. We have noticed that you have uploaded Supporting Information files, but you have not included a list of legends. Please add a full list of legends for your Supporting Information files after the references list.

3. As required by our policy on Data Availability, please ensure your manuscript or supplementary information includes the following:

A numbered table of all studies identified in the literature search, including those that were excluded from the analyses.

For every excluded study, the table should list the reason(s) for exclusion.

If any of the included studies are unpublished, include a link (URL) to the primary source or detailed information about how the content can be accessed.

A table of all data extracted from the primary research sources for the systematic review and/or meta-analysis. The table must include the following information for each study:

Name of data extractors and date of data extraction

Confirmation that the study was eligible to be included in the review.

All data extracted from each study for the reported systematic review and/or meta-analysis that would be needed to replicate your analyses.

If data or supporting information were obtained from another source (e.g. correspondence with the author of the original research article), please provide the source of data and dates on which the data/information were obtained by your research group.

If applicable for your analysis, a table showing the completed risk of bias and quality/certainty assessments for each study or outcome.  Please ensure this is provided for each domain or parameter assessed. For example, if you used the Cochrane risk-of-bias tool for randomized trials, provide answers to each of the signalling questions for each study. If you used GRADE to assess certainty of evidence, provide judgements about each of the quality of evidence factor. This should be provided for each outcome.

An explanation of how missing data were handled.

This information can be included in the main text, supplementary information, or relevant data repository. Please note that providing these underlying data is a requirement for publication in this journal, and if these data are not provided your manuscript might be rejected.

4. We note that your Data Availability Statement is currently as follows: " All data in available within the manuscript and its materials"

Please confirm at this time whether or not your submission contains all raw data required to replicate the results of your study. Authors must share the “minimal data set” for their submission. PLOS defines the minimal data set to consist of the data required to replicate all study findings reported in the article, as well as related metadata and methods (https://journals.plos.org/plosone/s/data-availability#loc-minimal-data-set-definition).

For example, authors should submit the following data:

- The values behind the means, standard deviations and other measures reported;

- The values used to build graphs;

- The points extracted from images for analysis.

Authors do not need to submit their entire data set if only a portion of the data was used in the reported study.

If your submission does not contain these data, please either upload them as Supporting Information files or deposit them to a stable, public repository and provide us with the relevant URLs, DOIs, or accession numbers. For a list of recommended repositories, please see https://journals.plos.org/plosone/s/recommended-repositories.

If there are ethical or legal restrictions on sharing a de-identified data set, please explain them in detail (e.g., data contain potentially sensitive information, data are owned by a third-party organization, etc.) and who has imposed them (e.g., an ethics committee). Please also provide contact information for a data access committee, ethics committee, or other institutional body to which data requests may be sent. If data are owned by a third party, please indicate how others may request data access.

**Additional Editor Comments (if provided):** **Reviewers' Comments:**

**Comments to the Author**

1. Does this manuscript meet PLOS Digital Health’s publication criteria?

Reviewer #1: Partly

Reviewer #2: Yes

Reviewer #3: Yes

**********

2. Has the statistical analysis been performed appropriately and rigorously?

Reviewer #1: No

Reviewer #2: N/A

Reviewer #3: Yes

**********

3. Have the authors made all data underlying the findings in their manuscript fully available (please refer to the Data Availability Statement at the start of the manuscript PDF file)?

The PLOS Data policy

Reviewer #1: No

Reviewer #2: Yes

Reviewer #3: Yes

**********

4. Is the manuscript presented in an intelligible fashion and written in standard English?

Reviewer #1: Yes

Reviewer #2: Yes

Reviewer #3: Yes

**********

Reviewer #1: This manuscript addresses an important and highly timely topic: the rapidly evolving landscape of artificial intelligence (AI) governance, reporting standards, editorial policies, and accountability frameworks in healthcare research and publication. The authors present a systematic narrative review that aims to identify, stratify, and synthesize governance instruments while proposing an evidence-tier framework intended to distinguish between formal reporting guidelines, consensus frameworks, emerging instruments, and policy statements.The manuscript is well written, generally well organized, and addresses a topic of considerable relevance to the readership of PLOS Digital Health. Major concerns: The abstract and Results state that 46 named frameworks were identified across six groups, and Table 1/Fig 3 report group sizes of G1=8, G2=5, G3=9, G4=8, G5=3, G6=8. These sum to 41, not 46. This discrepancy needs to be resolved and the count corrected throughout (abstract, Table 1 heading, Fig 3, and narrative text). A second major concern is the use of the relevance-scoring algorithm to prioritize records for screening. The review reports that over 30,000 records remained after deduplication, yet only 888 high-priority records proceeded to eligibility assessment. Consequently, the overwhelming majority of identified records were excluded prior to human review. Although the authors acknowledge this limitation, the manuscript does not provide sufficient evidence that the algorithm was validated or that potentially relevant frameworks were not systematically excluded. Information regarding algorithm development, validation procedures, sensitivity testing, and the likelihood of missed records is essential. Without such validation, it is difficult to assess whether the final sample accurately reflects the available literature. The evidence stratification framework represents the manuscript's principal methodological contribution. Firstly, the four governance phases and six thematic groups are presented as findings, but no method is described for how these categories were derived (e.g., inductive coding, consensus discussion, predefined taxonomy) or how disagreements were resolved. Furthermore, the process by which frameworks were assigned to tiers requires greater transparency. The criteria for tier assignment are described conceptually, but the manuscript does not indicate whether classifications were performed independently by multiple reviewers, whether disagreements were resolved through consensus, or whether any assessment of inter-rater reliability was conducted. Because several frameworks could reasonably be placed in different categories depending on interpretation, the reproducibility of the stratification process should be demonstrated more explicitly. Minor Issues: The abstract/title page describe the search as covering "January 2018 to May 2026," while Section 2.2 states searches were run "in April 2026, covering January 2018 to April 2026." These dates should be reconciled. The author name appears as "Monina Lino" on the title/metadata page but "Monica Lino" on the manuscript itself, please confirm correct spelling.The statement "All data in available within the manuscript and its materials" (note the typo) does not specify where within the manuscript or supplementary materials the underlying dataset (i.e., the full list of 102 included sources with extracted characteristics) can be found. Given that S4 File appears to contain exactly this dataset, the statement should explicitly name and reference it (e.g., "The full list of included sources and their extracted characteristics is available in S4 File")

Reviewer #2: This paper presents a narrative review of artificial intelligence

governance frameworks in healthcare. Given the explosion of interest

in health-care AI, particularly with respect to LLMs, this is a timely

topic. The volume of papers identified in this review provide further

evidence that a global perspective on the large amount of work that

has been done in developing disclosure frameworks is necessary. The

review is reasonably well conducted, with a few caveats. The

interpretations of the work are mostly reasonable, and the governance

tensions identified are interesting and appropriately suggest gaps in

existing work that might inform future endeavors.

I see no major flaws in the review strategy. The heuristic algorithm

used to filter the large number of references down to a manageable set

certainly introduces the possibility of bias, which is appropriately

acknowledged. It might be argued that this filtering step takes this

work out of the realm of "systematic" review, but I don't consider

this to be an important issue to stand on.

The six groups used in the summarization are frankly a bit puzzling

to me. Although four these group (1,3,4, and 6) are introduced in

figure 3.2, the complete list is not given in the main body of the

text - although it is in the abstract. That said, the groups are

somewhat puzzling. Why are "surgical reporting updates" (G5) separated

from "specialty-specific extensions" (G4)? No reasons are given. This

should be explained. Similarly, it seems puzzling that there is no

distinct separation between imaging frameworks (radiology/pathology)

and other non-imaging frameworks? These groups should be more clearly

defined and elucidated. Similarly, Table 1 would be much clearer if

textual labels for the groups were provided instead of group numbers.

The discussion of the four phases and the governance tensions raises

an interesting point. The timing of the final phases, occurring in

2023-2026, plays directly into the authority gradient: many of these

efforts might have been dealing with emerging understanding that was

simply too early to gain the perspective and consensus needed for the

more authoritative approaches. It's entirely appropriate for the

authors to point these shortcomings out as gaps. However, some

discussion of the timelines and the rapid evolution of the research

would be a welcome addition to the review.

Reviewer #3: REVIEW:

A complete review of AI governance frameworks must account for both international standards and national/regional policy regimes. On the standards side, ISO/IEC 42001 (the AI management system standard, analogous in structure to ISO/IEC 27001 for information security) and the NIST AI Risk Management Framework are foundational reference points — the former for organizations seeking a certifiable management-system approach, the latter for a voluntary, outcomes-based risk taxonomy (govern, map, measure, manage) that has been widely adopted as a common vocabulary even outside the US.

If the authors will exclude these popular frameworks (for not appearing in the search), it should explicitly state that such frameworks exist but were not found in the search.

**********

what does this mean?). If published, this will include your full peer review and any attached files.

**Do you want your identity to be public for this peer review?**  If you choose “no”, your identity will remain anonymous but your review may still be made public.

For information about this choice, including consent withdrawal, please see our Privacy Policy

Reviewer #1: No

Reviewer #2: **Yes:**  Harry Hochheiser

Reviewer #3: **Yes:**  Alvin Valeriano de Borja Marcelo

**********

**Figure resubmission:**

**Reproducibility:**

To enhance the reproducibility of your results, we recommend that authors of applicable studies deposit laboratory protocols in protocols.io, where a protocol can be assigned its own identifier (DOI) such that it can be cited independently in the future. Additionally, PLOS ONE offers an option to publish peer-reviewed clinical study protocols. Read more information on sharing protocols at https://plos.org/protocols?utm_medium=editorial-email&utm_source=authorletters&utm_campaign=protocols

# Author comment: Author response to Decision Letter 1

**DOI:** 10.1371/journal.pdig.0001783.r002

4 Aug 2026

Attachment Submitted filename: Response_to_Reviewers_PDIG-D-26-00944_FINAL.docx

# Peer review: Decision Letter 1

**DOI:** 10.1371/journal.pdig.0001783.r003

**Authors:** Hanieh Razzaghi, Amirhossein Zare

2 Sep 2026

Response to ReviewersRevised Manuscript with Track ChangesManuscript**Journal Requirements:** **Additional Editor Comments (if provided):** **Reviewers' Comments:**

**Comments to the Author**

Reviewer #1: All comments have been addressed

Reviewer #2: (No Response)

Reviewer #3: All comments have been addressed

**********

publication criteria?

Reviewer #1: Yes

Reviewer #2: Yes

Reviewer #3: Yes

**********

3. Has the statistical analysis been performed appropriately and rigorously?

Reviewer #1: Yes

Reviewer #2: N/A

Reviewer #3: Yes

**********

4. Have the authors made all data underlying the findings in their manuscript fully available (please refer to the Data Availability Statement at the start of the manuscript PDF file)?

The PLOS Data policy

Reviewer #1: Yes

Reviewer #2: Yes

Reviewer #3: Yes

**********

5. Is the manuscript presented in an intelligible fashion and written in standard English?

Reviewer #1: Yes

Reviewer #2: Yes

Reviewer #3: Yes

**********

Reviewer #1: The revised manuscript addresses a timely and important topic and has improved substantially in methodological transparency, internal consistency and presentation. The authors have responded constructively to the reviewers, corrected numerical and editorial inconsistencies, clarified the derivation of the six thematic groups and four governance phases, expanded the description of the relevance-scoring algorithm, and strengthened the data-availability statement.

However, several important methodological issues remain. The authors should validate the algorithm using an independent set of relevant records and a representative sample of below-threshold records. The manuscript should resolve the contradictory descriptions of whether manually identified EQUATOR records were independently retained or subjected to the same scoring threshold.

The derivation of the six thematic groups and four governance phases is now better explained, but these should be presented explicitly as an interpretive synthesis. More importantly, the governance domains displayed in Figure 2 are not described in the methodology. The manuscript does not explain whether these domains were predefined or derived inductively, how they were operationally defined, or how the ratings of “primary,” “moderate” and “minor” coverage were assigned. The unit of analysis, reviewers involved and process used to generate group-level ratings should be reported. Without this information, Figure 2 and the conclusions derived from it are not reproducible. Figure 1 also does not appear to be explicitly referenced in the main text and should be cited when presenting the search and selection results.

The evidence tiers should not be described as the “primary quality signal” without a formal quality-appraisal process. They classify instruments according to development pathway, publication status and institutional provenance, but do not directly measure methodological quality, validity, representativeness or implementability. They would be more accurately presented as developmental-maturity, governance-authority or instrument categories. The authors should also explain the reduction from 102 included sources in the original submission to 100 in the revision, reconcile the different analytical denominators, correct the inconsistent lead-author initials, provide an explicit disclosure concerning the use of AI tools in conducting or preparing the review, and moderate claims of comprehensiveness and global applicability given the restricted search sources and limited representation of non-English and LMIC literature.

Reviewer #2: Thanks for this revised submission. My major concerns have been addressed, leaving only a few minor points that would benefit from clarification.

1. The abstract and the text mention "the risk of epistemic equivalence". Later in Section 3.4.3 , the term "epistemic conflation" is used. "Epistemic equivalence" is a bit of an unsatisfactory term, as it implies that the risk lies in different items _being_ epistemically equivalent, which is not, what the paper rather implies. Rather, I think the authors are saying that there is a risk of "inappropriate perceptions of epistemic equivalence " - that readers will consider items to have the same degree of support even when they do not. I suggest either using the term "epistemic conflation" throughout or rephrasing to refer to the perception of epistemic equivalence.

2. Section 3.2 places the listing of the groups after the summary of those groups and their contents. This section might be clearer if the groups were moved to come before the summary.

3. This might be resolved during production, but in case it is not: the inclusion of a separate column for group in Table 1 takes up space without adding information, as the groups are given in separate table rows.

Reviewer #3: Thank you for covering this topic. It will serve as a good baseline for monitoring the progress of AI governance literature.

**********

what does this mean?). If published, this will include your full peer review and any attached files.

**Do you want your identity to be public for this peer review?**  If you choose “no”, your identity will remain anonymous but your review may still be made public.

For information about this choice, including consent withdrawal, please see our Privacy Policy

Reviewer #1: No

Reviewer #2: **Yes:**  Harry Hochheiser

Reviewer #3: No

**********

**Figure resubmission:**

**Reproducibility:**

To enhance the reproducibility of your results, we recommend that authors of applicable studies deposit laboratory protocols in protocols.io, where a protocol can be assigned its own identifier (DOI) such that it can be cited independently in the future. Additionally, PLOS ONE offers an option to publish peer-reviewed clinical study protocols. Read more information on sharing protocols at https://plos.org/protocols?utm_medium=editorial-email&utm_source=authorletters&utm_campaign=protocols

# Author comment: Author response to Decision Letter 2

**DOI:** 10.1371/journal.pdig.0001783.r004

5 Sep 2026

Attachment Submitted filename: PDIG-D-26-00944R2_Response_to_Reviewers.docx

# Editor report: Decision Letter 2

**DOI:** 10.1371/journal.pdig.0001783.r005

**Authors:** Hanieh Razzaghi, Amirhossein Zare

17 Sep 2026

Transparency, accountability, and scholarly integrity: a systematic narrative review of artificial intelligence governance frameworks in healthcare publication

PDIG-D-26-00944R2

Dear Dr Silva,

We are pleased to inform you that your manuscript 'Transparency, accountability, and scholarly integrity: a systematic narrative review of artificial intelligence governance frameworks in healthcare publication' has been provisionally accepted for publication in PLOS Digital Health.

Before your manuscript can be formally accepted you will need to complete some formatting changes, which you will receive in a follow-up email from a member of our team.

Please note that your manuscript will not be scheduled for publication until you have made the required changes, so a swift response is appreciated.

IMPORTANT: The editorial review process is now complete. PLOS will only permit corrections to spelling, formatting or significant scientific errors from this point onwards. Requests for major changes, or any which affect the scientific understanding of your work, will cause delays to the publication date of your manuscript.

If your institution or institutions have a press office, please notify them about your upcoming paper to help maximize its impact. If they'll be preparing press materials, please inform our press team as soon as possible -- no later than 48 hours after receiving the formal acceptance. Your manuscript will remain under strict press embargo until 2 pm Eastern Time on the date of publication. For more information, please contact digitalhealth@plos.org.

Thank you again for supporting Open Access publishing; we are looking forward to publishing your work in PLOS Digital Health.

Best regards,

Amirhossein Zare

Guest Editor

PLOS Digital Health

***********************************************************

**Additional Editor Comments (if provided):**

**Reviewer Comments (if any, and for reference):**
